# Examining the Omission of Dietary Quality Data in Glucagon-Like Peptide 1 Clinical Trials: A Scoping Review

**DOI:** 10.1016/j.advnut.2025.100491

**Published:** 2025-08-12

**Authors:** Demsina Babazadeh, Shawna Wyatt, Francene M Steinberg

**Affiliations:** Department of Nutrition, University of California Davis, Davis, CA, United States

**Keywords:** semaglutide, tirzepatide, dietary quality, clinical trials, scoping review

## Abstract

Injectable antiobesity medications (AOMs), including liraglutide, semaglutide, and tirzepatide, have demonstrated significant efficacy in promoting weight loss and improving glycemic control. However, the extent to which diet and food intake and related eating behaviors are assessed or reported in clinical trials of these agents remains unclear. This scoping review aimed to evaluate the presence and quality of dietary data, nutritional counseling, and related behavioral measures in randomized controlled trials of subcutaneous AOMs. A systematic literature search was conducted in MEDLINE-PubMed through September 2024, with a gap search completed December 2024. Eligible studies included randomized trials investigating liraglutide, semaglutide, or tirzepatide in humans. Studies were screened and extracted in Covidence, with 129 meeting inclusion criteria. Data extraction included AOM being studied, primary outcome, presence and type of nutritional or physical activity counseling, diet intake assessment and tools used, and eating behavior outcomes. Of 129 included studies, 54 evaluated liraglutide, 43 semaglutide, and 22 tirzepatide. Although 57 trials reported lifestyle modification as part of the intervention, 36 recorded diet quality and food intake. Among the 36 studies that collected dietary data, only 10 reported outcomes and half used single-time point assessments like ad libitum meals or buffets. Seventeen trials assessed food cravings or eating behavior using a variety of assessments. Across trials, there was minimal uniformity in outcome reporting, study duration, or counseling frequency, with most trials lacking detailed reporting on nutritional behavior components. Despite the central role of diet in weight regulation, most clinical trials involving AOMs fail to report meaningful diet quality or food intake data. The heterogeneity and underreporting of lifestyle components limit interpretability and generalizability of outcomes. Greater emphasis on standardizing and reporting dietary and behavioral measures is warranted to understand how AOMs interact with real-world nutrition behaviors and to inform comprehensive obesity care.

**Statement of significance**

This review identifies a critical gap in the clinical trial literature on injectable AOMs, where dietary intake data are rarely measured or reported despite consistent weight loss outcomes. The lack of dietary data limits our understanding of how these medications influence eating behaviors and undermines efforts to develop tailored nutrition guidance for long-term success.

## Introduction

Obesity, a complex and multifaceted chronic disease, has become one of the most prevalent health issues worldwide, affecting ∼650 million adults [1]. It is a major risk factor for a range of chronic conditions, including type 2 diabetes, metabolic disorders, hypertension, hyperlipidemia, stroke, and certain types of cancer. In the United States, clinical guidelines for the management of overweight and obesity primarily emphasize such as providing nutritional advice and physical activity modifications as first-line interventions [2]. However, these strategies are challenging to sustain long-term and often fail to achieve the substantial weight loss needed to effectively address the obesity epidemic [3].

To enhance weight loss outcomes, clinical guidelines now recommend the use of pharmaceutical interventions alongside lifestyle modifications for adults with overweight and obesity [4]. Liraglutide was the first generation of injectable antiobesity medications (AOMs) approved in the United States and belongs to the class of glucagon-like peptide-1 receptor agonists (GLP-1RAs). It is administered as a once-daily subcutaneous injection due to its shorter half-life. Semaglutide, a newer GLP-1RA, was initially approved by the United States FDA in 2017 for the treatment of type 2 diabetes [5]. Unlike liraglutide, semaglutide has a much longer half-life and is instead dosed once weekly. This extended duration improves convenience and may support adherence. Following extensive phase 3 clinical trials, particularly the Semaglutide Treatment Effects in People with Obesity (STEP) program, found that 2.4 mg semaglutide in combination with lifestyle modifications had clinically significant weight loss between 9.6% and 16.0% compared with placebo [[6], [7], [8], [9], [10], [11], [12], [13], [14]]; FDA approved semaglutide (Wegovy) for the treatment of obesity in 2021 [15].

Research behind the exact mechanism for this weight loss is still ongoing, but the current literature suggests it is linked to the activation of both the central and peripheral nervous systems. Specifically, GLP-1 RAs act on the GLP-1 receptors located in the hypothalamus related to food intake and energy balance, as well as indirect recruitment of the vagal afferent nerves in the gut and portal circulation leading to a reduction in appetite and decreased food intake [[16], [17], [18], [19]]. Tirzepatide, the most recent advancement in this class, is a once-weekly injectable dual agonist that targets both GLP-1 and glucose-dependent insulinotropic polypeptide (GIP) receptors. It has a half-life of ∼5 d, allowing for sustained receptor activation throughout the week. In addition to harnessing the appetite-suppressing effects of GLP-1, tirzepatide influences energy balance through GIP-mediated signaling in the brain and adipose tissue [20]. This dual mechanism produces a synergistic effect, resulting in enhanced efficacy for weight reduction [21]. Similar phase 3 clinical trials were conducted using weekly injections of tirzepatide for the treatment of obesity in adults [22]. The SURMOUNT trials (Study of Tirzepatide in Participants with Obesity or Overweight) evaluated the efficacy and safety of tirzepatide noting significant body weight (BW) loss percentages between 12.8% and 18.4% [[23], [24], [25], [26], [27], [28]] compared with placebo and bolstering even greater weight loss with tirzepatide than semaglutide in a head-to-head comparison, 20.2% compared with 13.7%, respectively [29]. For brevity, these medications will be referred to as GLP-1RAs hereafter.

The effectiveness of weight loss interventions has long relied on lifestyle modifications such as dietary strategies and physical activity modifications. A wide variety of diets, all centered on a reduction of energy intake, have been explored to promote weight loss and alleviate comorbidities associated with obesity. Notably, low-carbohydrate and low-fat diets have been the most extensively studied; however, neither has consistently demonstrated superior weight loss across the general population [30]. Instead, they have shown short-term weight loss across various diet compositions, dependent highly on the individual’s ability to maintain that level of energy reduction long term [31,32]. In an obesogenic environment, adherence to regimented dietary plans remains challenging, as biological and psychological factors pose a significant barrier to long-term weight loss and maintenance [3,26].

The FDA-approved prescribing information for semaglutide and tirzepatide explicitly states that these medications are intended to be used as an adjunct to lifestyle interventions, including a reduced-calorie diet and increased physical activity [33,34]. A recent joint advisory from the American College of Lifestyle Medicine, American Society for Nutrition, Obesity Medicine Association, and The Obesity Society outlined nutritional priorities intended to support the clinical use of GLP-1RAs [35]. This advisory highlighted gaps in widespread comprehensive lifestyle counseling, particularly emphasizing the necessity of nutrition and physical activity recommendations alongside GLP-1RA prescriptions. The advisory discussed considerations such as potential side effects, nutritional deficiencies, and protein requirements. Importantly, these considerations were derived primarily from broader contexts, including very low-calorie diets and bariatric surgery, and were not specific to GLP-1RA usage due to a lack of GLP-1RA-specific data [36]. The advisory advocated strongly for a multidisciplinary, team-based approach to optimize patient outcomes and called for more direct evidence from GLP-1RA clinical trials to guide nutritional counseling.

This is not the first advisory to address nutritional considerations for AOMs [37]. Existing recommendations, including those within the recent joint advisory, largely rely on indirect evidence due to the scarcity of dietary intake data explicitly collected from GLP-1RA clinical trials [38]. Despite emphasizing the importance for widespread coverage and referral for nutritional assessments and medical nutrition therapy conducted by registered dietitian nutritionists (RDNs), the specific nutrition recommendations for people using GLP-1RAs have predominantly referenced studies based on generalized dietary interventions, rodent models for dietary pattern and preference changes [39,40], controlled ad libitum buffet studies [[41], [42], [43]], or mathematical models estimating energy intake reductions [44]. Direct measurement of real-world dietary intake data from the extensive GLP-1RA clinical trials is notably absent from both guideline recommendations.

Therefore, we conducted a scoping review to survey, identify, and synthesize the existing literature on the relationship between the use of injectable AOMs, specifically the most recent and widely used FDA-approved injectable GLP-1RAs, such as liraglutide, semaglutide, and tirzepatide, and diet quality and food intake and food craving changes in humans. In this work, we aimed to answer the following questions to explore the extent and the nature of the existing evidence:

1. What is known about the relationship between the use of GLP-1RAs and changes in diet quality, food intake, or food cravings?
2. What gaps exist in the current literature, and how can future research better elucidate the role of dietary intake quality in the effectiveness of GLP-1RAs for obesity management?

## Methods

This review was conducted in accordance with the PRISMA-ScR guidelines [45]. The primary research question to be addressed was “What is known about the relationship between the use of GLP-1RAs and changes in diet quality, food intake, or food cravings?”

### Search strategy and study selection

A systematic literature search was conducted on June 12, 2024, with a subsequent gap search completed in December 2024 to ensure inclusion of the most recent studies. Searches were performed in the MEDLINE (via PubMed) database using a comprehensive set of terms to capture studies related to obesity, dietary intake, and injectable AOMs, particularly GLP-1 RA/dual-incretins. Search terms included variations of keywords such as “obesity,” “weight loss,” “diet quality,” “GLP-1,” “semaglutide,” “tirzepatide,” “liraglutide,” and branded drug names (e.g. Wegovy, Ozempic, Mounjaro, and Zepbound), along with filters for clinical trials, randomized controlled trials, and human studies. The complete search strategy is detailed in the Supplemental Methods. All retrieved citations were imported into Covidence systematic review software (Veritas Health Innovation, 2022), and duplicates were removed. Two reviewers (DB and SW) independently screened titles and abstracts, excluding studies that were not conducted in human populations or did not investigate 1 of the 3 currently FDA-approved subcutaneous AOMs: liraglutide, semaglutide, or tirzepatide. The screening process was conducted in 2 stages—initial title and abstract screening followed by full-text review—by 2 independent reviewers, with disagreements resolved through discussion. Full texts of the remaining studies were then assessed by (DB) for eligibility based on the inclusion criteria with oversight by another (FMS).

### Study selection: inclusion and exclusion criteria

Studies were eligible for inclusion if they met the following criteria: *1*) conducted in human participants, *2*) evaluated 1 or more of the currently FDA-approved subcutaneous AOMs—liraglutide, semaglutide, or tirzepatide—and *3*) reported findings from any type of clinical trial, including randomized controlled trials, open-label studies, or nonrandomized intervention trials. No restrictions were placed on participant age, sex, BMI, or health status, provided the study population included individuals with overweight or obesity. Studies were excluded if they: *1*) focused exclusively on other GLP-1 receptor agonists not currently approved for chronic weight management (eg, dulaglutide or exenatide) or primary intervention was oral/pill formulations only, *2*) were conducted in animals or in vitro, *3*) were not published in English, or *4*) were review articles, editorials, conference abstracts, post hoc analysis of already included trials, or study protocols without published results.

### Data extraction

From each included study, the following information was extracted: *1*) first author and year of publication; *2*) study design; *3*) GLP-1RA studied (liraglutide, semaglutide, or tirzepatide); *4*) sample size and population characteristics (e.g. age, BMI, and comorbidities); *5*) comparator group (e.g. placebo, lifestyle intervention, or alternative AOM); *6*) study duration; *7*) primary objective of the study and results; *8*) whether lifestyle modification counseling was reported; *9*) whether dietary intake was collected; *10*) the dietary assessment method used, if applicable; *11*) whether diet quality or intake patterns were reported; and *12*) whether eating behaviors such as food cravings and appetite were assessed. This structure was designed not only to document the presence of dietary assessment but also to highlight its absence and underreporting across trials.

## Results

### Study selection

**FIGURE 1:**
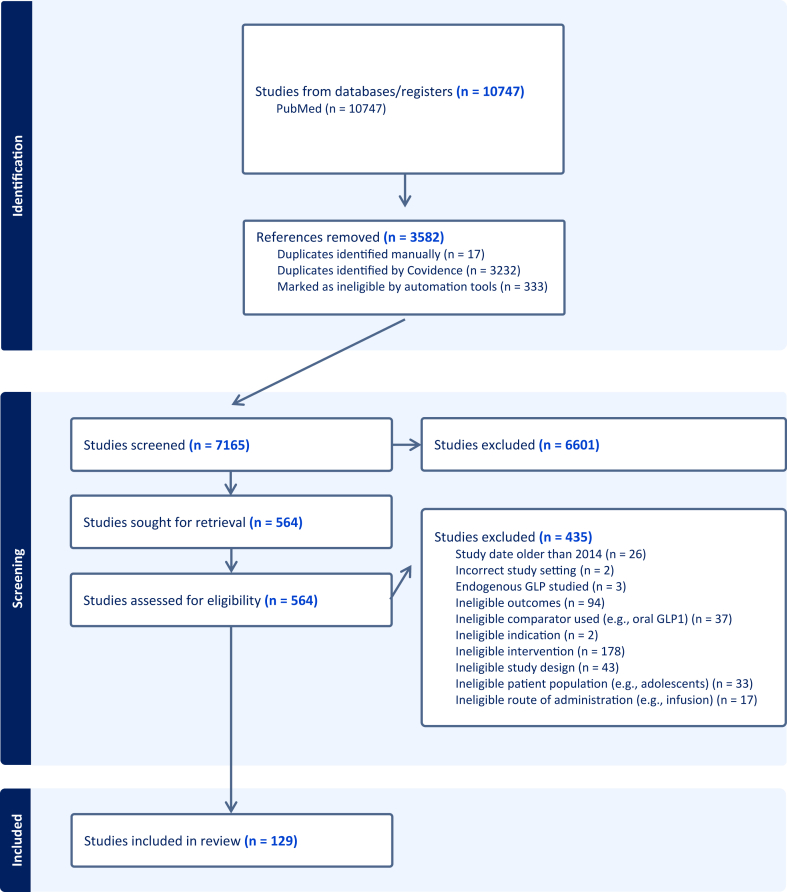
PRISMA flow diagram of search strategy and article selection process.

The search protocol initially identified 10,747 articles. After the removal of 17 duplicates identified manually and 3232 duplicates identified by Covidence, an additional 333 records were excluded by Covidence automation tools, which apply machine learning algorithms and keyword filters to flag studies deemed irrelevant based on title and abstract content. Abstracts from the remaining 7165 articles were screened for relevance, resulting in the exclusion of 6601 articles. Common reasons for full-text exclusion included use of ineligible interventions (e.g. endogenous GLP-1), ineligible comparators, nonrelevant outcomes (e.g. metabolic end points without dietary data), unsuitable study designs, or populations outside the scope of this review. Full-text review was conducted for the remaining 564 studies, of which 129 met all eligibility criteria and were included for data extraction. Figure 1 provides a PRISMA diagram illustrating the full screening and selection process.

Among the 129 studies included, the majority were phase 3 superiority trials investigating 1 or more of the 3 currently FDA-approved subcutaneous AOMs: liraglutide, semaglutide, and tirzepatide. Studies that featured these AOMs in head-to-head comparisons with non–FDA-approved AOM (including comparisons to oral formulations of approved GLP-1RAs) or other glucose-lowering agents, or medications such as sodium-glucose cotransporter-2 medications, were also included.

### GLP-1RA type by study

Fifty-four studies (41%) focused solely on liraglutide [43,[46], [47], [48], [49], [50], [51], [52], [53], [54], [55], [56], [57], [58], [59], [60], [61], [62], [63], [64], [65], [66], [67], [68], [69], [70], [71], [72], [73], [74], [75], [76], [77], [78], [79], [80], [81], [82], [83], [84], [85], [86], [87], [88], [89], [90], [91], [92], [93], [94], [95], [96], [97], [98]]. Forty-three studies (33%) examined semaglutide [[6], [7], [8], [9], [10], [11], [12], [13], [14],^42^,[99], [100], [101], [102], [103], [104], [105], [106], [107], [108], [109], [110], [111], [112], [113], [114], [115], [116], [117], [118], [119], [120], [121], [122], [123], [124], [125], [126], [127], [128], [129], [130], [131]], and 22 studies (17%) evaluated tirzepatide, the most recently FDA-approved AOM [[23], [24], [25], [26], [27], [28],^41^,[132], [133], [134], [135], [136], [137], [138], [139], [140], [141], [142], [143], [144], [145], [146]]. Ten studies (8%) evaluated 2 or more AOMs in a head-to-head comparison [[147], [148], [149], [150], [151], [152], [153], [154], [155], [156]], including 2 that compared injectable AOMs with oral counterparts [110,147].

### Primary outcomes

Forty-four studies focused on diabetes-related outcomes [[51], [52], [53], [54],^78^,^79^,^83^,^88^,[109], [110], [111], [112], [113], [114], [115], [116], [117], [118], [119], [120], [121], [122], [123],^128^,^129^,[136], [137], [138], [139], [140], [141], [142], [143], [144], [145], [146], [147], [148], [149], [150], [151],[154], [155], [156]], followed by 37 that evaluated AOM efficacy through changes in BW [[6], [7], [8],^10^,^12^,^13^,[23], [24], [25], [26], [27],[55], [56], [57], [58], [59],^62^,^66^,^67^,[70], [71], [72], [73], [74],^76^,^80^,^84^,^85^,^87^,^94^,^98^,[124], [125], [126], [127],^152^,^153^], 7 studies evaluated cardiovascular outcomes [14,48,49,92,104,105,131], 5 evaluated liver disease outcomes [47,91,102,103,135], and 6 were evaluating safety measures at different concentrations in various populations [10,99,100,130,132,134]. Six studies evaluated body–composition outcomes, such as changes in visceral adipose tissue [60,75], vO_2_ during cycle ergometry [69], and other body composition measures [41,61,86]. Sixteen studies evaluated some type of health measurement from gastric emptying [42,50,77,107], eating behaviors and food cue responses [9,43,64,81,82,89,93,97,98,106], and changes to health or weight-related quality of life scores [11,108]. Lastly, 2 studies measured changes to sleep apnea scores [28,68], 2 related to gallbladder emptying or volume [46,65], 2 related to kidney function [96,101], and 1 related to metabolites and lipid profiles [63].

### Lifestyle counseling

Of the 129 studies included in this review, 57 reported some form of lifestyle modification that involved dietary and/or physical activity counseling (Supplemental Table 1). Of these studies, 35 (61%) measured BW changes as the primary outcome [[6], [7], [8],^10^,^12^,^13^,[23], [24], [25], [26], [27],[55], [56], [57], [58], [59],^62^,^66^,^67^,[70], [71], [72], [73], [74],^76^,^80^,^84^,^85^,^87^,^94^,^124^,^125^,^127^,^152^,^153^]. A majority of these studies, 35 (61%), involved liraglutide [[43], [46], [47], [50], [51], [52], [55], [56], [57], [58], [59], [60], [61], [62], [63], [64], [66], [68], [70], [71], [72], [73], [74], [75], [76], [80], [81], [82], [83], [84], [85], [87], [91], [94], [95]] and 13 (23%) involved semaglutide [[6], [7], [8], [9], [10],^12^,^13^,^102^,^105^,^117^,^124^,^125^,^127^], and 3 (5%) were head-to-head comparisons of 2 or more GLP-1RAs [150,152,153]. The remaining 6 (10%) studies used tirzepatide as the AOM [[23], [24], [25], [26], [27], [28]].

### Responsible party for lifestyle counseling

Of the 57 studies that reported lifestyle modifications for nutrition, 32 documented using an RDN as the responsible party for delivering the intervention [[7], [8], [9], [10],^12^,^13^,[23], [24], [25],^27^,^46^,^47^,^51^,[55], [56], [57],^59^,[61], [62], [63], [64],^66^,[70], [71], [72],[74], [75], [76],^84^,^85^,^125^,^127^]. Within these 32 studies, only half explicitly named an RDN as the sole provider of the nutrition intervention [46,47,51,55,59,[61], [62], [63],[70], [71], [72],[74], [75], [76],^84^,^85^], whereas the other half allowed a similarly qualified health care professional to provide lifestyle modifications [[7], [8], [9], [10],^12^,^13^,[23], [24], [25],^27^,^56^,^57^,^64^,^66^,^125^,^127^]. Seventeen studies did not report who was responsible for the lifestyle counseling [6,28,43,52,58,60,68,73,82,87,91,94,95,102,105,117,150]. Five studies cited a qualified research staff, experienced practitioner, or qualified health care professional [26,83,124,152,153], 2 studies specifically named a behavioral psychologist with experience in obesity treatment [50,80], and 1 used an exercise physiologist [67].

### Dietary intake assessment methods

Among the included studies for this review, 36 collected dietary intake (Supplemental Table 1) Twenty-three studies used liraglutide [43,47,50,52,55,58,60,[62], [63], [64],[66], [67], [68],^70^,^72^,^73^,^76^,^77^,^80^,^84^,^91^,^93^,^97^], 10 involved semaglutide [[6], [7], [8], [9], [10],^42^,^106^,^124^,^125^,^127^], and 3 studied tirzepatide [24,25,41]. The predominant method of measuring intake was food logs or diaries. Participant-reported food diaries or food records were used by 26 studies [[6], [7], [8], [9], [10],^24^,^25^,^47^,^52^,^55^,^58^,^60^,[62], [63], [64],^66^,^68^,^72^,^73^,^76^,^84^,^93^,^97^,^124^,^125^,^127^], whereas 1 study used 24-h recalls [70], and 2 studies used food frequency questionnaires (FFQs) [67,91]. The remaining 7 studies employed direct dietary assessment, in which participants consumed a provided meal in a controlled setting with subsequent measurement of intake [[41], [42], [43],^50^,^77^,^80^,^106^].

### Dietary intake reporting

Of the 129 studies in this review, only 10 reported on diet intake data [42,43,50,70,77,80,91,93,97,106] (Table 1). Overall energy intake was reported in all these trials, whereas 3 studies reported additional intake changes for specific macronutrients [43,70,91]. None of these studies used comprehensive diet quality indices to report intake patterns, such as the Healthy Eating index 2020 [157,158], which measures adherence to the Dietary Guidelines for Americans, or the Alternate Healthy Eating Index [159], which assesses overall diet quality based on energy-adjusted nutrient and food group intake. Eight of the studies were done in liraglutide users [43,50,70,77,80,91,93,97], and the remaining 2 were in semaglutide users [42,106]. Two of these studies used food logs or diaries [93,97], 6 measured intake in single-time point assessments such as ad libitum meals or buffets [42,43,50,77,80,106], and only 1 study used either 24-h recalls [70] or FFQs [91] as assessment methods for intake data.

**TABLE 1:** Summary of studies reporting dietary outcomes in GLP-1RA trials by GLP-1RA type and author.

First author, year	GLP-1RA studied	Sample size and population characteristics	Nutritional counseling reported (Y/N)	Physical Activity Counseling reported (Y/N)	Dietary intake recorded (Y/N)	Dietary assessment method (if yes)	Reported diet quality or food pattern outcomes (Y/N)	Intake outcomes reported
Armstrong et al., 2016 [91]	Liraglutide	*N* = 52; 18–70 y of age; BMI: 25; histological evidence NASH based on liver biopsy	Y	Y	Y	Block FFQ	Y	Participants receiving liraglutide reported greater, although not statistically significant, reductions in total calorie intake (−291 ± 745 kcal; *P* = 0.12), protein (−14 g; 95% CI: −28, 0; *P* = 0.06), fat (−9 g; 95% CI: −24, 6; *P* = 0.22), and carbohydrates (−41 g; 95% CI: −96, 14; *P* = 0.14) than those receiving placebo, with no meaningful differences observed in caffeine or alcohol consumption between groups.
Farr et al., 2016 [97]	Liraglutide	*N* = 20; T2DM; HbA1c: >6.5%	N	N	Y	Food logs/diaries	Y	Participants in the liraglutide group reduced their total energy intake to 1434 ± 537 kcal/d by visit 4 vs. those in the placebo group to 1782 ± 542 kcal/d (*P* = 0.07).
Farr et al., 2019 [93]	Liraglutide	*N* = 28; BMI ≥ 30	N	N	Y	Food logs/diaries	Y	1782 ± 542 kcal/d in the placebo group vs. 1434 ± 537 kcal/d in the liraglutide group
Halawi et al., 2017 [50]	Liraglutide	*N* = 40; BMI ≥ 27 with 1 obesity-related comorbidity or BMI ≥ 30	Y	Y	Y	Ad libitum meal	Y	Calorie intake at the 16-wk buffet meal was lower with liraglutide than that with placebo [median: 554 kcal (IQR: 406–687 kcal) vs. 680 kcal (IQR: 513–1002 kcal)].
Quast et al., 2021 [43]	Liraglutide	*N* = 50; BMI ≤ 40; HbA1c: 6.5%–10.0%; T2DM for ≥3 mo	Y	Y	Y	45-min recorded ad libitum buffet	Y	The pooled analysis showed significant reductions from baseline in energy intake (−582.3 kj; 95% CI: −886.8, −277.8 kj; *P* = 0.0003), carbohydrates (−14.7 ± 4.6 g; *P* = 0.0015), protein (−5.2 ± 2.0 g; *P* = 0.011), and fat (−9.1 ± 2.8 g; *P* = 0.0032).
Sannaa et al., 2022 [80]	Liraglutide	*N* = 136; 18–65 y of age, BMI >30; lived within 125 miles of Mayo Clinic, Rochester, MN	Y	Y	Y	Ad libitum	Y	From baseline to the end of the study, total calorie intake decreased by 129.2 kcal (95% CI: −197.6, −23.2 kcal) in the placebo group and by 184.8 kcal (95% CI: −322.3, −69.4 kcal) in the liraglutide group.
Silver et al., 2023 [70]	Liraglutide	*N* = 88; 18–65 y of age; BMI ≥ 30; and having prediabetes defined by AMA; HbA1c: 5.7%–6.4%; fasting serum glucose: 100–125 mg/dL; and impaired glucose tolerance: 140–199 mg/dL after 2 h 75-g OGTT	Y	N	Y	24-h recalls	Y	Reduced intake by a mean of 300.0 ± 891.8 kcal/d (*P* = 0.007), no significant group differences observed for changes in total intake, energy reduction, or fat consumption; only changes in carbohydrate percentage and total and added sugar intake differed significantly by group.
van Can et al., 2014 [77]	Liraglutide	*N* = 49; BMI 30–40; stable BW < 5 kg × 90 d; fasting blood glucose < 7.0 mmol/l	N	N	Y	Ad libitum lunch	Y	Mean estimated energy intake during the ad libitum lunch was reduced by 588 and 568 kJ (∼16%) with liraglutide 1.8 mg (*P* = 0.002) and 3.0 mg (*P* = 0.003), respectively, vs. placebo.
Blundell et al., 2017 [106]	Semaglutide	*N* = 30; BMI: 30–45; HbA1c: <6.5%	N	N	Y	Standardized test meals and ad libitum meals, snack box during in-person stay	Y	24% reduction in total energy intake across all ad libitum meals throughout the day (−3036 kj; *P* < 0.0001).
Friedrichsen et al., 2021 [42]	Semaglutide	*N* = 72, BMI 30–45	N	N	Y	Ad libitum lunch	Y	Ad libitum energy intake was 35% lower with semaglutide vs. placebo (1736 kj vs. 2676 kj; *P* < 0.0001).

Abbreviations: AMA, American Medical Association; HbA1c, glycated hemoglobin; NASH, nonalcoholic steatohepatitis; OGTT, oral glucose tolerance test; T2DM, type 2 diabetes mellitus.

### Food eating behavior reporting

Seventeen studies reported outcomes related to food cravings or eating behaviors such as hunger, food preferences, and cravings (Table 2) [9,[41], [42], [43],^57^,^70^,^77^,^81^,^82^,^87^,^89^,^93^,^95^,^97^,^106^,^118^,^136^]. A wide variety of measurement tools were used in these studies and many included >1 assessment method. Most commonly, the visual analog scale was used to measure hunger, appetite, or taste changes in 10 studies [[41], [42], [43],^70^,^77^,^81^,^93^,^97^,^106^,^136^], whereas the Control of Eating Questionnaire (COEQ) was used in 5 studies [9,42,93,106,118], and 3 studies used the 3-factor eating questionnaire [57,87,95]. Two studies used functional magnetic resonance imaging (fMRI) imaging along with food cue presentation [89,97].

**TABLE 2:** Summary of GLP-1RA studies that reported food behaviors or craving outcomes by GLP1-RA type and author.

First Author, year	GLP-1RA studied	Sample size and population characteristics	Comparator group	Study duration	Primary outcome measured	Nutritional counseling reported (Y/N)	Physical Activity Counseling reported (Y/N)	Food behavior assessed (Y/N)	Food behavior assessment method (if yes)	Cravings outcome results
Farr et al., 2016 [97]	Liraglutide	*N* = 20; T2DM HbA1c: >6.5%	1:1 Liraglutide 1.8 mg vs. placebo with 3-wk washout	4 wk each	Change between highly desirable vs. less desirable food cues	N	N	Y	fMRI signals with food cues, VAS	There were no changes in brain activations to food cues during the fed state. Liraglutide-induced reductions in circulating leptin were significantly associated with increased postprandial fullness, as measured by VAS ratings (ρ = −0.544, *P* = 0.016).
Farr et al., 2019 [93]	Liraglutide	*N* = 28; BMI ≥ 30	Liraglutide 3.0 mg/d vs. placebo	5 wk + 1-wk washout	fMRI responses to food cue	N	N	Y	VAS and COEQ	When controlled for BMI/weight, liraglutide increased activation of the right orbitofrontal cortex in response to food cues (*P* < 0.016, corrected for multiple comparisons).
Jensterle et al., 2015 [95]	Liraglutide	*N* = 36; women; stable BW ≤ 5% BW change × 6 mo; premenopausal; BMI ≥ 30; taking metformin 2000 mg for ≥6 mo	Liraglutide 1.2 mg	12 wk	Eating behavior	Y	Y	Y	TFEQ	Liraglutide significantly reduced uncontrolled eating (36.8 → 19.6) and emotional eating scores (49.9 → 28.5; *P* < 0.001), with no change in cognitive restraint.
Kadouh et al., 2020 [81]	Liraglutide	*N* = 40; BMI ≥ 27 with 1 obesity-related comorbidity or BMI ≥ 30	1:1 Liraglutide 3.0 mg vs. placebo	16 wk	Appetite and taste scores	Y	Y	Y	Standardized drink test (maximum tolerated volume), VAS	Study participants in the liraglutide group demonstrated significant reduction in the desire to eat something sweet, salty, savory, or fatty and an increase in perceived fullness.
Lundgren et al., 2021 [57]	Liraglutide	*N* = 195; 18–65 y of age; BMI: 32–43. Participants followed a low-calorie diet of 800 kcal/d × 8 wk. If lost ≥5% of their baseline BW, then they were randomly assigned	1:1:1:1 Ratio exercise + placebo, liraglutide + usual activity, exercise + liraglutide, placebo. Participants stratified by age and sex.	52 wk	Change in BW	Y	Y	Y	Food preference questionnaire (Leeds Food preference Questionnaire) and subjective appetite sensation, TFEQ	Decreased appetite 37% in the liraglutide group, 33% in the combination group, 8% in the exercise-only group, and 4% in the placebo group.
McElroy et al., 2024 [87]	Liraglutide	*N* = 60; BMI ≥ 27 with ≥1 obesity-related comorbidity or BMI ≥ 30; stable bipolar disorder type 1 or type 2 based on Diagnostic and Statistical Manual of Mental Disorders; Fourth Edition; receiving stable psychotropic regimen for past 90 d	Liraglutide 3.0 mg/d vs. placebo	40 wk	Change in BW	Y	Y	y	TFEQ and BES	Participants receiving liraglutide had significantly greater reductions in TFEQ hunger subscale score vs. placebo. There were no significant differences between groups in changes in eating psychopathology in the TFEQ cognitive restraint or inhibition scores.
Quast et al., 2021 [43]	Liraglutide	*N* = 50; BMI ≤ 40; HbA1c: 6.5%–10.0%; T2DM for ≥3 mo	Liraglutide 1.8 mg/d vs. lixisenatide 20 μg/d	10 wk	Appetite and energy intake	Y	Y	Y	Appetite for sweet, salty, hearty, or fatty meals using a VAS	Patients on liraglutide reported reduced perceived eating capacity (4.60 ± 0.58 cm vs. 6.29 ± 0.66 cm; *P* < 0.006) and lower anticipated eating pleasure (4.87 ± 0.61 cm vs. 6.33 ± 0.62 cm; *P* < 0.01) vs. placebo, both of which remained significant when controlling for changes in weight or BMI.
Robert et al., 2015 [82]	Liraglutide	*N* = 44; binge-eaters (BES > 18)	Liraglutide 1.8 mg + diet and exercise vs. control (diet and exercise only)	12 wk	BES score, ghrelin levels	Y	Y	Y	Ghrelin biomarker to measure hunger	AUC ghrelin increased with liraglutide treatment (855.6–1002.8 ng/mL·min; *P* = 0.018), consistent with compensatory responses seen in diet-induced weight loss. Ghrelin levels significantly increased, which have the potential to diminish the weight loss effects of liraglutide beyond the intervention.
Silver et al., 2023 [70]	Liraglutide	*N* = 88; 18–65 y of age; BMI ≥ 30; and having prediabetes defined by AMA; HbA1c: 5.7%–6.4%; fasting serum glucose: 100–125 mg/dL; impaired glucose tolerance: 140–199 mg/dL after 2 h 75-g OGTT	2:1:1 Liraglutide 1.8 mg vs. sitagliptin 100 mg/d, vs. calorie-restriction diet	14 wk	Change in BW	Y	N	Y	Hunger VAS during overnight-fasted metabolic testing visits	The changes from baseline to end of study in participants’ ratings of feeling hungry, feeling satisfied, feeling full, or how much participants perceived they could consume at their next meal were not significantly different among treatment groups. Participants in the liraglutide group rated their feeling of fullness higher (*P* = 0.003) and their rating of how much they could consume lower (*P* = 0.02) at the end of the intervention period.
Ten Kulve et al., 2016 [89]	Liraglutide	*N* = 20; BMI ≥ 26; HbA1c: 6.0%–8.5%; T2DM treated with metformin with or without sulfonylureas; right-handed; Caucasian; stable BW of <5% change in past 90 d	Liraglutide 1.8 mg/d vs. insulin glargine	36 wk	BOLD fMRI signal change from baseline	N	N	Y	10-point Likert, fMRI after standardized meal with visual food cues	Patients treated with liraglutide showed decreased responses to food pictures in insula and putamen (*P* ≤ 0.02) vs. insulin glargine with 10 d of treatment. Liraglutide enhanced the satiating effect of meal intake on responses in putamen and amygdala (*P* ≤ 0.05). But differences seen between liraglutide and insulin glargine were not sustained after 12 wk.
vanCan et al., 2014 [77]	Liraglutide	*N* = 49, BMI 30–40, stable BW <5 kg × 90 d, fasting blood glucose <7.0 mmol/L	Liraglutide 1.8 mg vs. 3.0 mg vs. placebo	5 wk + 2 d in clinic then cross over with 6–8-wk washout	Gastric emptying after standard meal	N	N	Y	Appetite VAS during inpatient stays after 5 wk	Liraglutide 1.8 mg and 3.0 mg significantly increased postprandial satiety, fullness, and overall appetite scores vs. placebo, indicating reduced appetite. Hunger ratings also improved postprandially, whereas thirst and nausea ratings showed minimal differences between groups.
Blundell et al., 2017 [106]	Semaglutide	*N* = 30; BMI: 30–45; HbA1c: <6.5%	1:1 Semaglutide or placebo	12 wk × 2 crossover with 5–7-wk wash out	Ad libitum energy intake during a lunch meal (5 h after standard breakfast)	N	N	Y	VAS, COEQ, and LFPQ	Semaglutide was associated with less hunger and food cravings, better control of eating and a lower preference for high-fat foods and nonsweet foods vs. placebo (*P* = 0.0016). Differences between treatments in explicit liking for other food categories were not significant. Postprandial increases from fasting VAS ratings showed greater increases in satiety with semaglutide vs placebo; however, differences in the overall incremental appetite suppression score were not significant.
Dwibedi et al., 2024 [118]	Semaglutide	*N* = 239; HbA1c ≥ 6.5%; T2DM ongoing metformin; features of SIDD or SIRD	1:1 Semaglutide or dapagliflozin in addition to baseline metformin	24 wk	% Change in HbA1c	N	N	Y	COEQ	Semaglutide participants with SIRD reported less hunger, less difficulty in resisting food cravings and less cravings for starchy foods as compared with participants with SIDD.
Friedrichsen et al., 2021 [42]	Semaglutide	*N* = 72; BMI: 30–45	1:1 Semaglutide 2.4 mg vs. placebo	20 wk	Gastric emptying after standardized test meal	N	N	Y	VAS and COEQ	Semaglutide reduced hunger and prospective food consumption and increased fullness and satiety vs. placebo (all *P* < 0.02). COEQ indicated better control of eating and fewer/weaker cravings with semaglutide vs. placebo (*P* < 0.05).
Wharton et al., 2023 [9]	Semaglutide	*N* = 174; BMI ≥ 30 or 27 with 1 obesity-related comorbidity	1:1 Semaglutide 2.4 mg vs. placebo	104 wk	Association between changes in COEQ domain scores and BW	Y	Y	Y	COEQ	At 104 wk, scores for desire to eat salty and spicy food, cravings for dairy and starchy foods, difficulty in resisting cravings, and control of eating were significantly reduced with semaglutide vs. placebo (all *P* < 0.05).
Heise et al., 2023 [41]	Tirzepatide	*N* = 117; T2DM ≥ 6 mo, treated with lifestyle advice or stable metformin	3:3:2 Tirzepatide 15 mg, semaglutide 1 mg, or placebo	28 wk	Measurements of body composition, appetite, and energy intake were performed as secondary assessments	N	N	Y	Fasted VAS ratings of hunger, satiety, fullness, and prospective food consumption were completed, and a composite of the 4 scores was used to calculate an overall appetite score	At wk 28, appetite reduced from baseline, as reflected by higher overall appetite scores, with tirzepatide and semaglutide (*P* < 0.001) but not placebo (*P* = 0.241). Tirzepatide and semaglutide significantly reduced appetite vs. placebo. Appetite scores and energy intake reductions did not differ between tirzepatide and semaglutide.
Yabe et al., 2022 [136]	Tirzepatide	*N* = 48; age > 20 y; BMI ≥ 23; HbA1c: 7.0%–10.0%; T2DM; diet and exercise control or monotherapy antidiabetic agent	1:1:1:1 Tirzepatide 5 mg, 10 mg, 15 mg, vs. dulaglutide 0.75 mg	52 wk	Pharmacodynamic effects of medications on postprandial metabolic characteristics of appetite and AUC_0–6 h_ after dose for plasma glucagon, serum glucose, insulin, C-peptide, and triglycerides	N	N	Y	Appetite was assessed for ≤6 h following each meal tolerance test, with fullness and hunger assessed via VAS	Participants receiving tirzepatide reported dose-dependent improvements in appetite measures, with fullness scores increasing by +89.4 mm (SE: 33.8), +63.2 mm (SE: 29.1), and +94.9 mm (SE: 32.8) for the 5-, 10-, and 15-mg doses, respectively, compared with +47.1 mm (SE: 22.8) with dulaglutide 0.75 mg. Correspondingly, hunger scores decreased by −56.8 mm (SE: 33.2), −78.5 mm (SE: 28.9), and −65.2 mm (SE: 31.4) for tirzepatide, vs. −27.3 mm (SE: 22.4) for dulaglutide.

Abbreviations: AMA, American Medical Association; BES, Binge Eating Scale; BOLD, blood oxygen level–dependent; BW, body weight; COEQ, Control of Eating Questionnaire; fMRI, functional magnetic resonance imaging; HbA1c, glycated hemoglobin; OGTT, oral glucose tolerance test; SIDD, severe insulin-deficient diabetes; SIRD, severe insulin-resistant diabetes; T2DM, type 2 diabetes mellitus; TFEQ, 3-Factor Eating Questionnaire; VAS, visual analog scale.

## Discussion

This scoping review provides an overview of 129 clinical trials evaluating FDA-approved injectable AOMs—liraglutide, semaglutide, and tirzepatide—with a focus on how diet quality, food intake, and lifestyle counseling are addressed. As the role of these medications expands beyond their original usage for glycemic control, trials increasingly explore their impact on weight management and obesity-related comorbidities. Understanding how lifestyle components are integrated into these studies is essential to inform comprehensive, real-world obesity care.

### Diet quality and food intake reporting and lifestyle counseling

Despite the central role of nutrition in obesity and metabolic health, relatively few trials incorporated structured dietary assessments or lifestyle counseling. Of the 129 trials reviewed, only 36 (28%) mentioned collecting some form of dietary intake assessment (Supplemental Table 1), and only 10 of those reported specific diet quality and food intake outcomes (Table 1). All 10 studies observed reductions in energy intake, although not all reached statistical significance.

Notably, semaglutide, evaluated for intake outcomes in only 2 of the 43 semaglutide trials included in this review, produced substantial and statistically significant reductions in energy intake—demonstrating a 24% reduction across ad libitum meals [106] and a 35% reduction during a single lunch period (1736 kJ compared with 2676 kJ; *P* < 0.0001) [42]. Liraglutide similarly reduced estimated intake during a lunch meal by ∼16% (588 and 568 kJ for 1.8 and 3.0 mg, respectively; *P* = 0.002 and *P* = 0.003, respectively) [77], and 1 study using a controlled buffet setting reported significant reductions from baseline in energy intake (−582.3 kJ; *P* = 0.0003), carbohydrates (−14.7 ± 4.6 g; *P* = 0.0015), protein (−5.2 ± 2.0 g; *P* = 0.011), and fat (−9.1 ± 2.8 g; *P* = 0.0032) [43].

Although these findings underscore the anorectic effects of GLP-1RAs, ad libitum test meals are limited by their design as single time-point assessments conducted in highly controlled settings, which may not reflect habitual eating behaviors or real-world conditions. They may not capture habitual intake or long-term behavioral adaptation. In contrast, studies using self-reported food logs, 24-h recalls, or FFQs offered a more flexible and ecologically valid approach, albeit with greater variability and potential reporting bias [160]. For example, using food logs and diaries, liraglutide reduced average energy intake to ∼1434 kcal/d, compared with 1782 kcal/d in the placebo group, with a trend toward significance (*P* = 0.07) [93,97]. In a study using 24-h recalls, a mean reduction of 300 ± 891.8 kcal/d during liraglutide treatment (*P* = 0.007) was reported [70]. Armstrong et al. [91] reported nonsignificant reductions in protein, fat, and carbohydrates, using FFQs suggesting trends that align with other trials but possibly underpowered for significance with only 52 people included in the trial. Together, these data suggest pharmacologic modulation of food intake, although the methods of collection and reporting remain inconsistent across trials. This heterogeneity limits direct comparisons and highlights the need for standardized assessment tools in future trials.

Notably, 8 of the 10 studies that did collect and report on diet quality and food intake were conducted with liraglutide [43,50,70,77,80,91,93,97], whereas only 2 semaglutide studies reported diet outcomes [42,106], and none were identified for tirzepatide. This highlights a substantial gap in dietary intake data—including energy, macronutrient composition, and dietary patterns—for newer weekly injectables, particularly semaglutide and tirzepatide. Half of the studies that recorded intake also included lifestyle counseling, and all were in liraglutide users [43,50,70,80,91]. Of note, a single study by Blundell et al. [106] did not report lifestyle counseling, however, demonstrated substantial reductions in energy intake, albeit on a single time point ad libitum lunch meal (e.g. −3036 kJ/d with semaglutide). Without information linking diet quality and food intake to structured behavior change, our ability to attribute changes to combined pharmacologic and behavioral intervention is limited.

### Eating behavior

Nearly all 17 studies that evaluated GLP-1RAs for eating behavior or craving changes (Table 2) reported reductions in hunger or improvements in satiety and fullness, regardless of the specific medication used. These outcomes were assessed using validated tools: the visual analog scale, which measures subjective sensations of hunger and satiety [161]; the COEQ, which evaluates frequency and intensity of cravings, control over eating, and cravings for specific food types [162]; the 3-factor eating questionnaire, which assesses cognitive restraint, disinhibition, and hunger susceptibility [163]; and fMRI, which visualizes brain activity in reward and appetite-regulating regions in response to food cues [164]. Liraglutide was associated with reduced hunger and improved eating behavior scores [81,87,95], semaglutide lowered cravings in both general and metabolically distinct populations (e.g. severe insulin-resistant diabetes compared with severe insulin-deficient diabetes) [118], and tirzepatide showed dose-dependent improvements in hunger and fullness ratings, outperforming dulaglutide in direct comparisons [136].

A few studies used objective measures: Farr et al. [97] linked reduced leptin to increased postprandial fullness (ρ: –0.544, *P* = 0.016), whereas another study by Farr et al. [93] found increased OFC activation with liraglutide (*P* < 0.016). Ten Kulve et al. [89] reported reduced insula and putamen activity after 10 d of liraglutide (*P* ≤ 0.02), with increased satiation signals in the amygdala and putamen (*P* ≤ 0.05), although not sustained at 12 wk. Robert et al. [82] found that AUC ghrelin increased with liraglutide, suggesting a compensatory hormonal effect.

Despite reductions in appetite scores, changes in actual behavior or intake were not always observed. Silver et al. [70] reported increased fullness and lower perceived intake with liraglutide (*P* = 0.003; *P* = 0.02), but no changes in hunger or satisfaction compared with sitagliptin or diet. Wharton et al. [9] found semaglutide reduced cravings for specific foods and improved eating control using COEQ over 104 wk. Jensterle et al. [95] noted reductions in uncontrolled and emotional eating scores with liraglutide (*P* < 0.001), whereas cognitive restraint remained unchanged. These findings highlight the utility of capturing nuanced behavioral adaptations that may not be detected through appetite ratings alone.

### Studies without dietary intake data reporting

Of the 93 studies that did not include dietary intake assessment, most prioritized cardiometabolic or organ-specific end points, such as glycemic control, liver histology, and cardiovascular function. These were often large-scale, long-duration phase 2 or 3 trials enrolling thousands of participants. Even among the studies that included lifestyle interventions only about half tracked dietary intake, limiting our ability to determine whether observed outcomes were due to medication, behavioral changes, or both.

The majority of these 93 studies without dietary intake assessments—including the SUSTAIN (semaglutide) and SURPASS (tirzepatide) trials—were well-powered and long in duration, yet did not report lifestyle counseling or collect any dietary intake data. Their end points focused on glycemic control, such as hemoglobin A1c. SUSTAIN trials enrolled between 388 and 3297 participants and spanned 30–104 wk (Supplemental Table 2), and SURPASS trials included 316–2002 adults with diabetes over similar timeframes (Supplemental Table 3), yet neither were structured to evaluate drivers of weight change.

In contrast, the STEP trials—designed to evaluate weight loss—incorporated structured lifestyle counseling across all 10 trials, reporting weight loss, ranging from 9.6% to 17.4% (Supplemental Table 4). Counseling was delivered monthly, either by a dietitian or similarly qualified health care professional, with guidance to follow a 500-kcal/d deficit diet and engage in ≥150 min of physical activity per week. Dietary self-monitoring was encouraged, but diet quality or intake outcomes were not reported, except in 1 substudy that used COEQ to report changes in cravings [9].

SURMOUNT trials evaluating tirzepatide mirrored the STEP design, including similar counseling structures. Participants receiving 15 mg tirzepatide lost 15.0%–22.5% of their baseline weight over 72–88 wk (Supplemental Table 5). Only SURMOUNT 2 [24] and SURMOUNT 3 [25] reported use of diet and activity logs, which were reviewed but not analyzed or reported as outcomes.

Given the behavioral complexity of achieving such as weight loss, the absence of dietary intake data in these trials limits our understanding of how GLP-1RAs influence eating behaviors. Although self-reported dietary data face challenges such as recall bias and underreporting [160], they remain the most scalable option in real-world trials. Digital tools like smartphone applications, photograph-assisted logs, or web-based recalls can reduce burden and improve feasibility [165]. Landmark nutrition studies like the Nurses’ Health Study [166], European Prospective Investigation into Cancer and Nutrition [167], and Framingham Heart Study [168] demonstrate the value of self-reported intake data. Inclusion of such tools in future AOM trials may provide insights into evolving dietary behaviors, including changes in intake patterns, food cravings, and taste preferences during treatment.

### Measurements of eating behavior

Incorporating validated craving assessments in AOM trials is particularly important given the established role of GLP-1RAs in modulating eating behaviors such as, appetite, craving changes, and food preferences. Obesity has been associated with taste alterations, potentially related to sensory aspects of eating or the food reward response system [169]. Preclinical and clinical studies have demonstrated that GLP-1 and its analogs cross the blood–brain barrier and act on central nervous system structures involved in appetite regulation, including the hypothalamus and mesolimbic dopamine system [40,170,171]. Animal models have shown that GLP-1 receptor activation in the ventral tegmental area and nucleus accumbens can reduce food-motivated behavior and preference for palatable foods, implicating these pathways in the suppression of hedonic eating [81,172,173]. Human neuroimaging studies have corroborated this mechanism. For example, researchers have demonstrated that earlier GLP1-RAs—for example, exenatide—attenuates activation of brain regions associated with food reward in response to high-calorie visual food cues [165,174]. Similarly, Farr et al. [175] found that liraglutide significantly reduced fMRI activation in the parietal cortex and insula in response to food stimuli, independent of weight loss [175], however, some of these results reflect only short-term administration, and long-term impacts on brain reward systems is limited [89].

### Responsible party for lifestyle counseling

One critical issue that emerged across trials offering lifestyle counseling was the inconsistent use of RDNs as the sole provider of nutrition lifestyle counseling. RDNs possess specialized expertise in medical nutrition therapy, dietary assessment, and behavioral counseling, making them uniquely qualified to deliver effective lifestyle interventions in obesity management [127]. Despite this, 16 clinical trials reviewed referred ambiguously to an RDN or similarly qualified health care professional (Supplemental Table 1), which introduces uncertainty about the expertise of the individual providing the lifestyle counseling intervention. Trials such as STEP-3 [8] and SURMOUNT-3 [25] explicitly used structured intensive behavioral therapy [176]—including meal replacements and counseling delivered only by RDNs—with semaglutide and tirzepatide, respectively. STEP-3 reported greater weight loss outcomes (16.0%) [8] than STEP-1 (14.9%) [6], which had a similar trial design and population but did not include intensive behavioral therapy, underscoring the importance of a nutrition expert guiding lifestyle modifications. As noted in the joint advisory, however, a persistent challenge remains—the inconsistent insurance coverage for medical nutrition therapy—limiting access to RDNs whose specialized training in nutrition care is recommended, yet whose impact remains largely undocumented in trial outcome reporting [35].

The Look AHEAD (Action for Health in Diabetes) trial demonstrated the long-term value of intensive lifestyle intervention; with >5000 participants, those who received structured support led by RDNs showed significantly improved dietary quality after 1 y compared with controls without dietary counseling [177]. This reinforces the importance of integrating clearly defined, expert-led nutrition strategies alongside pharmacological treatments to ensure patients develop the self-management skills necessary to sustain health improvements—particularly in cases of medication discontinuation due to cost, insurance coverage, side effects, or other barriers. Standardizing RDN inclusion within AOM trials would enhance the rigor of dietary interventions and improve both clinical relevance and translational impact of study findings.

Additionally, understanding the sustainability of weight loss while on GLP-1RAs is critical, particularly given that patients often regain approximately two-thirds of the weight initially lost within 1 y of discontinuation [178]. Although these medications are intended for long-term use in chronic disease management [179], real-world adherence is challenged by numerous factors—including high out-of-pocket costs, insurance coverage changes, side effects, adverse events, and access disparities [180]. The high rates of discontinuation further underscore the need for RDN involvement, both to support sustainable behavioral change and to provide continuity of care during medication interruptions. On this point, both the findings of this review and the recommendations outlined in the joint advisory [35], strongly align.

### Limitations

Although this scoping review comprehensively mapped lifestyle and dietary reporting in injectable AOM trials, it is not without limitations. The analysis was limited to published articles and did not include unpublished protocols, gray literature, or all supplemental materials, where additional intervention details may reside. Furthermore, non–English-language trials were excluded, which may limit generalizability. As a scoping review, this analysis did not assess study quality or risk of bias but rather aimed to describe the scope and nature of dietary and lifestyle assessment across trials.

### Implications for future research

Unlike the narrative review by Christensen et al. [38], which provides expert commentary on potential dietary strategies for patients prescribed GLP-1 and GIP/GLP-1RAs, and the joint advisory from the American College of Lifestyle Medicine, the American Society for Nutrition, the Obesity Medicine Association, and the Obesity Society [35], which offers practice-oriented guidance, this scoping review systematically catalogs clinical trials that report on actual dietary intake, craving behavior, and assessment methods. This distinction allows for a focused evaluation of empirical evidence gaps across trials of GLP-1-based AOMs.

This review underscores persistent gaps in the reporting of dietary intake and behavioral interventions in AOM trials, despite the central role these factors play in mediating metabolic outcomes. Although weight loss and glycemic improvements were consistently reported, the behavioral mechanisms driving these changes—particularly those related to diet quality and food intake, cravings, and physical activity—remain under characterized. Just as pharmacologic efficacy must be interpreted within the context of biological variability, the impact of AOMs should be examined within the behavioral and nutritional environments in which they are administered. To advance the field and provide scientific rigor, future trials must adopt a more robust and transparent framework for designing, delivering, and reporting lifestyle interventions. Key recommendations include the consistent use of validated dietary assessment tools, greater methodological standardization, comprehensive reporting of diet intake and food-related behaviors, and strategic inclusion of qualified nutrition professionals (RDNs) (Table 3). A deeper integration of these domains will be essential to fully realize the therapeutic potential of AOMs and translate clinical trial findings into equitable, sustainable, and practice-ready models of obesity care.

**TABLE 3:** Key recommendations for future studies.

Key recommendations for future studies
Essential
• Report behavioral outcomes: Include outcomes related to dietary intake and eating behavior—not just weight loss or medication adherence. Accurate reporting of actual intake data are foundational to understanding behavioral mechanisms and interpreting trial results.
Preferred
• Specify lifestyle intervention protocols: Clearly define the frequency, delivery format, and credentials of those providing counseling (e.g. registered dietitians), to support reproducibility and clarify the dose and fidelity of behavioral support.
Ideal
• Use validated assessment tools: Incorporate standardized, validated tools to assess diet, cravings, hunger, and physical activity (e.g. Healthy Eating Index-2020, Alternate Healthy Eating Index, Control of Eating Questionnaire, and visual analog scale) to improve measurement quality and cross-study comparability.
• Collect contextual variables: Capture social determinants of health such as food insecurity, cultural food practices, and access to healthy foods, which may influence behavioral response and medication effectiveness across diverse populations.

### Conclusions

Although the primary goal of most GLP-1RA trials was to evaluate safety and efficacy, this review found that only 10 of 129 studies (∼8%) reported dietary intake or dietary patterns—despite the clear role these medications play in influencing appetite and eating behavior. Although dietary change was not the intended focus of a majority of these studies, the exclusion of these data limits the ability to contextualize the observed weight loss and undermines efforts to develop specific nutritional recommendations for patients on GLP-1RAs.

Additionally, lifestyle intervention reporting across trials lacked consistency. Measures related to food cravings, appetite, physical activity, and adherence were either inconsistently applied or absent altogether. The use of validated nutrition assessment tools was uncommon, reducing comparability across studies. Broader behavioral and environmental influences, including food insecurity, cultural dietary preferences, and access to nutritious foods, were also rarely assessed. As GLP-1RAs become more widely used in clinical practice, incorporating these contextual variables will be important to understanding who benefits most—and why—and to inform equitable, long-term obesity treatment strategies.

## Author contributions

The authors’ responsibilities were as follows—DB, FMS: planned the research; DB: conducted the initial literature search; DB, SW: screened the studies for inclusion with DB doing the final data extraction with oversight from FMS; DB: was responsible for the writing of the manuscript with review and final edits by FMS; and all authors have read and approved the final version of the manuscript.

## Funding

This study was supported by USDA CA-D-NTR-6316-H (FS) UC Davis College of Agricultural and Environmental Science Henry A. Jastro graduate research award (to DB).

## Conflict of interest

The authors report no conflicts of interest.
