# Supplementary material for: Examining the Omission of Dietary Quality Data in Glucagon-Like Peptide 1 Clinical Trials: A Scoping Review
Source: Adv Nutr. 2025 Aug 12;16(10):100491. doi: 10.1016/j.advnut.2025.100491 (PMC12592236; doi:10.1016/j.advnut.2025.100491)
Supplement: Multimedia component 1 [file mmc1.docx]

**Supplemental materials**

**Examining the Omission of Dietary Quality Data in GLP-1 Clinical Trials: A Scoping Review**

Demsina Babazadeh MPH, RDN^1^, Shawna Wyatt BS^1^ & Francene M. Steinberg PhD, RDN^1*^

**Supplemental Methods: Search Strategy**

A comprehensive literature search was conducted using PubMed to identify studies evaluating GLP-1 receptor agonists and diet-related outcomes in the context of weight loss and obesity. The date of the last search was 12/6/2024. The search strategy included combinations of terms related to obesity, weight loss, dietary intake, and specific GLP-1 receptor agonists (semaglutide, liraglutide, tirzepatide). Filters for clinical trials, randomized controlled trials, and human studies were applied where applicable. All trials were filtered for the year 2014 and beyond.

Below is the full list of search strings used:

| **Search Term #** | **Search String** |
| --- | --- |
| **1** | (((((obes*) AND (weightloss)) OR (diet quality)) OR (diet)) AND (medications) NOT (exercise) AND ((clinicaltrial[Filter] OR randomizedcontrolledtrial[Filter]) AND (humans[Filter]))) AND (GLP1*) |
| **2** | ((((obes*) AND (weightloss)) OR (diet quality)) OR (diet)) AND (medications) AND ((clinicaltrial[Filter] OR randomizedcontrolledtrial[Filter]) AND (humans[Filter])) |
| **3** | (((((obes*) OR (overweight)) AND (weightloss)) AND (GLP1*)) OR (semaglutide)) OR (tirzepatide) |
| **4** | ((obes*) OR (overweight)) AND (GLP1*) OR (semaglutide) OR (tirzepatide) |
| **5** | ((obes*) OR (overweight)) AND (GLP1*) |
| **6** | (((obes*) AND (GLP1*)) NOT (insulin)) NOT (diabetes) |
| **7** | ((((obes*) AND (weightloss)) OR (diet quality)) OR (diet)) AND (medications) NOT (exercise) |
| **8** | ((((obes*) AND (weightloss)) OR (diet quality)) OR (diet)) AND (medications) |
| **9** | (((GLP1*) OR (antiobesity medication)) OR (anti-obesity*)) AND (obes*) |
| **10** | (((GLP1*) OR (antiobesity medication)) OR (anti-obesity)) AND (obes*) |
| **11** | (((((((GLP1) OR (GLP1*)) OR (Semaglutide)) OR (wegovy)) OR (Tirzepatide)) OR (Zepbound)) OR (mounjaro)) OR (Ozempic) |
| **12** | (((((((GLP*) OR (GLP1 receptor agonist)) OR (Semaglutide)) OR (wegovy)) OR (Tirzepatide)) OR (Zepbound)) OR (mounjaro)) OR (Ozempic) |
| **13** | ((semaglutide) OR (tirzepatide) OR (liraglutide)) AND (weightloss) AND (diet) |
| **14** | ((semaglutide) OR (tirzepatide)) AND (weightloss) AND (diet) |
| **15** | ((semaglutide) OR (tirzepatide)) AND (diet quality) |
| **16** | ((semaglutide) OR (tirzepatide)) AND (food intake) |
| **17** | ((semaglutide) OR (tirzepatide)) AND (food) |
| **18** | ((semaglutide) OR (tirzepatide)) AND (diet) |
| **19** | (((((semaglutide) OR (tirzepatide)) AND (diet)) OR (food)) |
| **20** | (((((semaglutide) OR (liraglutide)) OR (tirzepatide)) ) AND (diet)) OR (food) |
| **21** | (((Semaglutide) OR (Liraglutide)) OR (Tirzepatide)) AND (weightloss) |
| **22** | tirzepatide trials AND obes* |

These searches were designed to capture a wide range of clinical trials and studies involving GLP-1 receptor agonists and their interaction with dietary intake or food behavior. Duplicates were removed manually prior to screening.

Supplemental Table 1 Summary of Studies Reporting Lifestyle Counseling, Dietary intake collection and frequency by GLP-1RA type and Author

| **Author & year** | **GLP-1RA studied** | **Sample size & population characteristics** | **Intervention** | **Study duration** | **Primary outcome measured** | **Nutritional counseling reported (Y/N)** | **Physical activity counseling reported (Y/N)** | **Responsible party for lifestyle modification** | **Frequency of lifestyle modification** | **Dietary intake collected (Y/N)** | **Dietary intake assessment method (if applicable)** | **Frequency of intake collection (if applicable)** |
| --- | --- | --- | --- | --- | --- | --- | --- | --- | --- | --- | --- | --- |
| Armstrong et al, 2016 (91) | Liraglutide | n=52, 18-70 yrs of age, BMI 25, histological evidence NASH based on liver biopsy | Liraglutide 1.8 mg vs. Placebo | 60 wk | Resolution of definite NASH with no worsening in fibrosis from baseline to end of treatment | Y | Y | Unknown | Unknown | Y | Block FFQ | Baseline, at the end of treatment, and 12 wks after the end of treatment |
| Blackman et al, 2016 (68) | Liraglutide | n=359, 18-64 yrs of age, stable BW < 5% change in past 90 days, BMI >/= 30, moderate AHI 15-19.9 events/hour or severe AHI >/= 30.0 events with no continuous positive airway pressure | 1:1 liraglutide 3.0 mg vs. Placebo | 32 wk | Change in AHI after 32 wk | Y | Y | Unknown | Monthly | Y | Food logs/diaries | Every 5-8 wks |
| Chao et al, 2019 (66) | Liraglutide | n=150, age 21-70, BMI 30-55, prior lifetime wt loss effort with diet and exercise | IBT only, IBT + liraglutide 3.0mg/d vs. IBT + liraglutide 3.0 mg/d + 12 wk of 1000-1200 kcal/day meal replacements | 52 wk | %change BW | Y | Y | RDN or similarly qualified health care professional | 21 sessions over 52 wks | Y | Food logs/ diaries | Unknown |
| Coppin et al, 2023 (64) | Liraglutide | n=66, BMI 30-45, stable BW</= 5% change within 90 days, right-handed, non-smoker, and were enrolled in a structured multidisciplinary patient education wt loss program at the hospital | Liraglutide 3.0 mg + lifestyle counseling vs. Placebo + lifestyle counseling | 16 wk | Change of bold signal (%) in brain regions involved in the reward system | Y | Y | RDN or similarly qualified health care professional | Unknown | Y | Food logs/diaries | Unknown |
| Davies et al, 2015 (62) | Liraglutide | n=846, BMI >/= 27, HbA1c 7.0-10.0%, T2DM taking oral anti-diabetic agents | 2:1:1 liraglutide 3.0 mg, liraglutide 1.8 mg, vs. Placebo | 52 wk | %change BW baseline to 56 wk and proportion individuals losing >/= 5% baseline BW to 56 wk, and proportion of individuals losing >/=10% baseline BW to 56 wk | Y | Y | RDN | Monthly | Y | Food logs/diaries | Monthly |
| Elkind-Hirsch et al, 2022 (85) | Liraglutide | n=88, BMI>30, polycystic ovary syndrome | 2:1 liraglutide 3.0 mg vs. Placebo | 32 wk | Change in BW | Y | Y | RDN | At start of visit only | N | n/a | n/a |
| Farr et al, 2016 (97) | Liraglutide | n=20, T2DM HbA1c >6.5% | 1:1 liraglutide 1.8 mg vs. Placebo with 3 wk washout | 4 wk each | Change between highly desirable vs. Less desirable food cues | N | N | n/a | n/a | Y | Food logs/diaries | Baseline & end of study |
| Farr et al, 2019 (93) | Liraglutide | n=28, BMI >/= 30 | Liraglutide 3.0 mg/d vs. Placebo | 5 wk + 1 wk washout | fMRI responses to food cues | N | N | n/a | n/a | Y | Food logs/diaries | Baseline & end of study |
| Feng et al, 2019 (73) | Liraglutide | n=85, BMI 20-38, HbA1cc 7.0-14%, T2DM no hypoglycemic drug usage in 90 days, diagnosed with NAFLD no medications or history of autoimmune liver | 1:1:1 metformin, liraglutide, vs. Gliclazide | 24 wk | %change in BW and body composition | Y | Y | Unknown | 7 visits over 24 wks | Y | Food logs/diaries | At each visit* |
| Garvey et al, 2020 (56) | Liraglutide | n=396, BMI >/= 27, stable BW (</= 5kg change in 90 days), HbA1c 6.0-10%, T2DM on stable basal insulin and </= 2 oral anti-diabetic agents | 1:1 liraglutide 3.0 mg vs. Placebo | 56 wk | %change BW baseline to 56 wk and proportion individuals losing >/= 5% baseline BW to 56 wk | Y | Y | RDN or similarly qualified health care professional | 23 individual or group counseling sessions during the 56 wk period | N | n/a | n/a |
| Grannell et al, 2021 (61) | Liraglutide | n=78, BMI > 35 with at least 1 obesity-related comorbidity | 1:1 lifestyle intervention alone vs. Lifestyle intervention + liraglutide 3.0 mg | 16 wk | Change in fat free mass comparing the different treatments of liraglutide 3.0 mg plus standard care vs. standard care alone | Y | Y | RDN | Every 2-4 wks | N | n/a | n/a |
| Gudbergsen et al, 2021 (59) | Liraglutide | n=156, 18-74 yrs of age, BMI>/= 27, symptomatic knee OA, early-to moderate knee OA changes | Upon successfully achieving a wt loss of minimum 5% at wk 0, continued with a tapering dietary intervention for 8 wk (wk 0 to 8) 1:1 liraglutide 3.0 mg vs. Placebo | 52 wk | Change in BW from baseline to 52 wk and change in Knee Injury and OA Outcome Score pain subscale from baseline to 52 wk | Y | Y | RDN | wk –8 to 0: weekly;  wk 0 to 8: biweekly;  wk 8 to 52: no sessions; | N | n/a | n/a |
| Halawi et al, 2017 (50) | Liraglutide | n=40, BMI >/= 27 with 1 obesity-related comorbidity or BMI >/= 30 | 1:1 liraglutide 3.0 mg vs. Placebo | 16 wk | Change in gastric emptying (delay relative to baseline) of solids t1/2 of radiolabeled meal to empty from the stomach at 5 wk and 16 wk | Y | Y | Psychologist | Monthly | Y | Ad libitum buffet | Baseline & end of study |
| Jensterle et al, 2015 (95) | Liraglutide | n=36, women, stable BW </= 5% change x 6 months, premenopausal, BMI >/= 30, taking metformin 2000 mg for at least 6 months | Liraglutide 1.2 mg | 12 wk | Eating behavior | Y | Y | Unknown | Beginning of study | N | n/a | n/a |
| Jensterle et al, 2017 (94) | Liraglutide | n=30, women, Type A polycystic ovary syndrome by American society for reproductive medicine and European society of human reproduction and embryology; Rotterdam criteria, BMI>/=30 | Metformin +liraglutide 1.2 mg (low dose) vs. Liraglutide 3.0 mg (high dose) | 12 wk | Change in BW | Y | N | Unknown | Beginning of study | N | n/a | n/a |
| Kadouh et al, 2020 (81) | Liraglutide | n=40, BMI >/= 27 with 1 obesity-related comorbidity or BMI>/= 30 | 1:1 liraglutide 3.0 mg vs. Placebo | 16 wk | Appetite and taste scores | Y | Y | Psychologist | Monthly | Y | Ad libitum buffet | Baseline & end of study |
| Khoo et al, 2019 (47) | Liraglutide | n=30, Asian, BMI > 30, waist circumference >/= 90 cm in men, >/= 80 cm in women, diagnosed with NAFLD and steatohepatitis with liver fat fraction >/= 5.5% on MRI | 1:1 diet-exercise group vs. Liraglutide 3.0 mg | 26 wk | Reductions in wt, liver fat content and serum transaminase levels from baseline to 26 wk | Y | Y | RDN | Every 2-4 wks | Y | Food logs/diaries | Every 2-4 wks |
| Kim et al, 2014 (51) | Liraglutide | n=68, ages 40-70 yrs old, BMI 27-40, with prediabetes | 1:1 liraglutide 1.8 mg (daily) vs. Placebo | 14 wk | Effects on beta cell function of wt loss augmented by liraglutide compared with wt loss alone | Y | Y | RDN | Weekly for the first 4 wks then bimonthly | N | n/a | n/a |
| Le roux et al, 2017 (52) | Liraglutide | n=2254, BMI >/= 30, BMI >/= 27 with obesity related comorbidities | 2:1 liraglutide 3.0 mg vs. Placebo | 2 yrs + 12 wks in prediabetes | Time to onset of diabetes as the primary endpoint | Y | Y | Unknown | Monthly | Y | Food logs/diaries | Every 2^nd^ month |
| Lundgren et al, 2021 (57) | Liraglutide | n=195, 18-65 yrs of age, BMI 32-43 | Participants followed a low-calorie diet of 800 kcal/day x 8 wks. If lost >/= 5% of their baseline BW were randomly assigned, in a 1:1:1:1 ratio exercise + placebo, liraglutide + usual activity, exercise + liraglutide, placebo. | 52 wk | Change in BW from randomization to 52 wk | Y | Y | Dietitian/scientific professional | 12 sessions over 52 wks | N | n/a | n/a |
| Matikainen et al, 2019 (55) | Liraglutide | n=23, BMI 27-40, HbA1c 6.0-9.0%, T2DM, stable treatment, triglycerides 1.0-4.0 mmol/l, LDL-cholesterol < 4.5 mmol/l | 1:1 liraglutide 1.8 mg vs. Placebo | 16 wk | Mean wt loss baseline to 16 wk | Y | N | RDN | Weekly | Y | Food logs/diaries | Baseline & end of study |
| McElroy et al, 2024 (87) | Liraglutide | n=60, BMI>/= 27 with at least 1 obesity-related comorbidity or BMI >/= 30, stable Bipolar Disorder Type 1 Or Type 2 Based On Diagnostic And Statistical Manual Of Mental Disorders, Fourth Edition, receiving stable psychotropic regimen for past 90 days | Liraglutide 3.0 mg vs. Placebo | 40 wk | Change in BW | Y | Y | Unknown | Baseline, visit 4, visit 6, and visits 10 to 15 | N | n/a | n/a |
| Mensberg et al, 2016 (67) | Liraglutide | n=33, BMI >25, T2DM treated with diet and or metformin, HbA1c 7-11%, sedentary lifestyle (<150 min/wk activity) | Exercise + liraglutide 1.8 mg vs. Exercise + placebo | 16 wk | Change in BW | N | Y | Trained exercise physiologists | Weekly | Y | FFQ | Baseline, midway, and end of study |
| Neeland et al, 2021 (60) | Liraglutide | n=185, age >/= 35, BMI >/= 30, or BMI>/=27 with prevalent Metabolic Syndrome (National Cholesterol Education Program Adult Treatment Panel iii Criteria) | 1:1 liraglutide 3.0 mg vs. Placebo | 46 wk | efficacy of liraglutide vs. Placebo in reducing VAT measured by MRI | Y | Y | Unknown | Unknown | Y | Food logs/diaries | Run-in phase and periodically throughout study |
| Nexoe-larsen et al, 2018 (46) | Liraglutide | n=52, 18-64 yrs of age, GMI>/= 27 with stable BW <3kg change in 90 days, acceptable gallbladder volume quality on screening | 1:1 liraglutide 3.0 mg vs. Placebo | 12 wk | Gallbladder volume measured by ultrasonography, fasted and at 14 predefined timepoints within the 240-min following test meals | Y | Y | RDN | 5 sessions over 12 wk | N | n/a | n/a |
| Pantalone et al, 2021 (71) | Liraglutide | n=200, BMI>/= 30, enrolled in employer health plan for which they were recruited | 1:1 wt management program vs. wt management program + liraglutide | 52 wk | %change in BW | Y | Y | RDN | Monthly | N | N | n/a |
| Peradze et al, 2019 (63) | Liraglutide | n=20, BMI >30 or >/27 with comorbidities of obesity | 1:1 liraglutide 3.0 mg vs. Placebo | 5 wk | Effects on metabolites, lipid, and lipoprotein profiles | Y | Y | RDN | Weekly | Y | Food logs/diaries | Weekly |
| Pi-Sunyer et al, 2015 (58) | Liraglutide | n=3731, BMI >/=30, or >/=27 with untreated dyslipidemia or hypertension | 2:1 liraglutide 3.0 mg vs. Placebo, | 56 wk | %change BW baseline to 56 wk and proportion individuals losing >/= 5% baseline BW to 56 wk, and proportion of individuals losing >/=10% baseline BW to 56 wk | Y | Y | Unknown | Monthly | Y | Food logs/diaries | Every 2^nd^ month |
| Quast et al, 2021 (43) | Liraglutide | n=50, BMI </= 40, HbA1c 6.5-10.0%, T2DM for at least 3 months | Liraglutide 1.8 mg/d vs. Lixisenatide 20 μg/d | 10 wk | Appetite and energy intake | Y | Y | Unknown | Unknown | Y | 45 min recorded ad libitum buffet | After an overnight fast on the preceding day, before and on the last day of the treatment period. |
| Robert et al, 2015 (82) | Liraglutide | n=44, binge-eaters (BES> 18) | Liraglutide 1.8 mg + diet and exercise vs. Control (diet and exercise only) | 12 wk | BES score, ghrelin levels | Y | Y | Unknown | Every 6 wks | N | n/a | n/a |
| Sannaa et al, 2022 (80) | Liraglutide | n=136, 18-65 yrs of age, BMI >30, lived within 125 miles of Mayo Clinic, Rochester, MN | Liraglutide 3 mg vs. Placebo | 16 wk | Wt loss | Y | Y | Psychologist | Monthly | Y | Ad libitum | Baseline & end of study |
| Santilli et al, 2017 (75) | Liraglutide | n=40, BMI >/= 30, impaired glucose tolerance, impaired fasting glucose, T2DM <12 mo according to American Diabetes Association Guidelines, could be treated with diet therapy + metformin up to 3000 mg/d | 1:1 liraglutide 1.8mg vs. Lifestyle counseling | 60 wk | Change in VAT after achievement of wt loss target | Y | Y | RDN | Unknown | N | n/a | n/a |
| Silver et al, 2023 (70) | Liraglutide | n=88, 18-65 yrs of age, BMI >/= 30, and having pre-diabetes defined by AMA, HbA1c 5.7-6.4%, fasting serum glucose 100-125 mg/dl, impaired glucose tolerance 140-199 mg/dl after 2 hours 75g OGTT | 2:1:1liraglutide 1.8 mg vs, sitaglipitin 100 mg/d, vs. Calorie restriction diet | 14 wk | Change in BW | Y | N | RDN | Every 2 wks | Y | 24-hr recalls x 3 days | Baseline and end of study |
| Takeshita et al, 2022 (83) | Liraglutide | n=120, HbA1c >/= 8.0%, T2DM, treated by diet therapy and/or oral anti-diabetic agent for >/= 12 wk | 0.9 mg liraglutide x 24 wk vs., insulin degludec/day x 12 wks followed by liraglutide x 12 wk | 24 wk | Changes in the levels of fasting blood glucose, 1,5-anhydroglucitol and HbA1c | Y | Y | Experienced practitioner | Unknown | N | n/a | Unknown |
| Tronieri et al, 2019 (76) | Liraglutide | n=45, BMI>/= 27, stable BW </= 5kg BW change in 90 days | Liraglutide 3.0 mg + placebo vs. Liraglutide 3.0 mg + phentermine 15.0 mg | 12 wk | %change in BW | Y | Y | RDN | Every 3-4 wks | Y | Food logs/diaries | Unknown |
| Tronieri et al, 2020 (72) | Liraglutide | n=282, BMI >/=30, stable BW </= 5 kg change in 90 days | 1:1 liraglutide 3.0 mg + IBT vs placebo +IBT | 52 wk | %change in BW | Y | Y | RDN | 23 visits over 52 wks | Y | Food logs/diaries | At each visit* |
| Tronieri et al, 2020 (74) | Liraglutide | n=113, BMI >/=30, stable BW </= 5 kg BW change in 90 days | 1:1 liraglutide 3.0 mg + IBT vs. Placebo +IBT | 52 wk | %change in BW | Y | Y | RDN | Weekly for 4 sessions, every-other-wk for 10 sessions, every 4 wks for 7 sessions | N | n/a | n/a |
| VanCan et al, 2014 (77) | Liraglutide | n=49, BMI 30-40, stable BW <5kg x 90 days, fasting blood glucose <7.0 mmol/l | Liraglutide 1.8 mg vs. 3.0 mg vs. Placebo | 5 wk + 2 days in clinic then cross over with 6-8 wk washout | Gastric emptying after standard meal | N | N | n/a | n/a | Y | Ad libitum lunch | End of study |
| Wadden et al, 2019 (84) | Liraglutide | n=150, age 21-70 yrs of age, BMI 30-55, prior lifetime wt loss effort with diet and exercise | IBT only, IBT + liraglutide 3.0mg/d vs. IBT + liraglutide 3.0 mg/d + 12 wk of 1000-1200 kcal/day meal replacements | 52 wk | Change BW | Y | Y | RDN | Every 2-4 wks; wk 0 to 8: biweekly; wk 8 to 52: no sessions | Y | Food logs/diaries | 21 sessions over 52 wks |
| Bliddal et al, 2024 (12) | Semaglutide | n=407, BMI >/= 30, clinical diagnosis of Knee OA According To The American College Of Rheumatology Criteria With Pain Related To Knee OA On Western Ontario and McMaster Universities OA Index Pain Score >/= 40 | 2:1 semaglutide 2.4 mg vs. Placebo | 68 wk | %change in BW and the change in Western Ontario And McMaster Universities OA index pain score from baseline to 68 wk | Y | Y | RDN or similarly qualified health care professional | Weekly | N | n/a | n/a |
| Blundell et al, 2017 (106) | Semaglutide | n=30, BMI 30-45, HbA1c <6.5% | 1:1 semaglutide or placebo | 12 wk x 2 crossover with 5-7 wk wash out | Ad libitum energy intake during a lunch meal (5 hrs after standard breakfast) after 12 wk | N | N | n/a | n/a | Y | Standardized test meals and ad libitum meals, snack box during in-person stay | At end of study |
| Davies et al, 2021 (7) | Semaglutide | n=1210, BMI >/= 27, T2DM | 1:1:1, 2.4 mg, 1.0 mg., vs. Placebo | 68 wk | %change BW baseline to 68 wk & loss of at least 5% BW at 68 wk | Y | Y | RDN or similarly qualified health care professional | Monthly | Y | Food logs/diaries | Monthly |
| Friedrichsen et al, 2021 (42) | Semaglutide | n=72, BMI 30-45 | 1:1 semaglutide 2.4 mg vs. Placebo | 20 wk | Gastric emptying after standardized test meal | N | N | n/a | n/a | Y | Ad libitum lunch | At end of study |
| Garvey et al, 2022 (125) | Semaglutide | n=304, BMI >/= 30 or 27 with 1 obesity-related comorbidity | 1:1 semaglutide 2.4 mg vs. Placebo | 104 wk | %change BW baseline to 104 wk & loss of at least 5% BW at 104 wk | Y | Y | RDN or similarly qualified health care professional | Monthly | Y | Food logs/diaries | Monthly* |
| Ingersen et al, 2023 (117) | Semaglutide | n=31, 40-70 yrs of age, BMI>28, wt stable <2kg change in 6 months, T2DM with moderately preserved β-cell | 12 wk of aerobic training vs semaglutide x 20 wk before 12 wk of aerobic training (with continued semaglutide) | 32 wk | Change in insulin secretory capacity with training, evaluated by a 2-stepped hyperglycemic (20 and 30 mm) clamp | N | Y | Unknown | 3 times a wk x 12 wks | N | n/a | n/a |
| Kadowaki et al, 2022 (10) | Semaglutide | n=401, BMI >/= 35 or 27 with >/= 2 obesity-related comorbidity | 4:1:2:1 semaglutide 2.4 mg vs. Placebo or semaglutide 1.7 mg vs. Placebo | 68 wk | %change BW baseline to 68 wk & loss of at least 5% BW at 68 wk | Y | Y | RDN or similarly qualified health care professional | Monthly | Y | Food logs/diaries | Monthly* |
| Lincoff et al, 2023 (105) | Semaglutide | n=17604, >45 yrs of age, BMI >/= 27, established cardiovascular disease | 1:1 semaglutide 2.4 mg vs. Placebo | 59 months (mean duration of follow up was 39.8 months) | Cardiovascular efficacy end point was a composite of death from cardiovascular causes, nonfatal myocardial infarction, or nonfatal stroke, assessed in a time-to-first-event analysis | Y | Y | Unknown | Weekly | N | n/a | n/a |
| Loomba et al, 2023 (102) | Semaglutide | n=71, histological evidence of NASH and Kleiner f4 according to the NASH clinical research network classification based on single liver biopsy | 2:1 semaglutide 2.4 mg vs. Placebo, stratified by diabetes status | 48 wk | Proportion of patients with an improvement in liver fibrosis of one stage or more on biopsy (using the NASH classification) without worsening of NASH after 48 wk | Y | Y | Unknown | Baseline, at four follow-up visits from wk 12 until end of treatment | N | n/a | n/a |
| McGowan et al, 2024 (13) | Semaglutide | n=138, BMI >/= 30, and prediabetes having at least 1: HbA1c 6.0-6.4% or fasting plasma glucose 5.5-6.9 mmol/l | 2:1 semaglutide 2.4 mg vs. Placebo | 52 wk | %change BW baseline to 52 wk and proportion of participants who reverted to normoglycemia | Y | Y | RDN or similarly qualified health care professional | 9 visits over 52 wks | N | n/a | n/a |
| Mu et al, 2024 (127) | Semaglutide | n=375, BMI >/= 30 or 27 with 1 obesity-related comorbidity, T2DM if diagnosed >/= 180d prior and on stable treatment | 2:1 semaglutide 2.4 mg vs. Placebo | 44 wk | Change in BW | Y | Y | RDN or similarly qualified health care professional | Monthly | Y | Food logs/diaries | Monthly |
| Rubino et al, 2021 (124) | Semaglutide | n=902, BMI >/= 30 or 27 with 1 obesity-related comorbidity | All open label semaglutide until wk 20; 2:1 semaglutide 2.4 mg vs. Placebo after wk 20 | 68 wk | %change BW wk 20 to wk 68 | Y | Y | Experienced practitioner | Monthly | Y | Food logs/diaries | Monthly* |
| Wadden et al, 2021 (8) | Semaglutide | n=611, BMI >/= 30 or 27 with 1 obesity-related comorbidity | 2:1 semaglutide 2.4 mg vs. Placebo | 68 wk | %change BW baseline to 68 wk & loss of at least 5% BW at 68 wk | Y | Y | RDN or similarly qualified health care professional | Every 2 wks | Y | Food logs/diaries | Every 2 wks |
| Wharton et al, 2023 (9) | Semaglutide | n=174, BMI >/= 30 or 27 with 1 obesity-related comorbidity | 1:1 semaglutide 2.4 mg vs. Placebo | 104 wk | Association between changes in COEQ domain scores and BW | Y | Y | RDN or similarly qualified health care professional | Monthly | Y | Food logs/diaries | Monthly |
| Wilding et al, 2021 (6) | Semaglutide | n=1961, BMI >/= 30 or 27 with 1 obesity-related comorbidity | 2:1 semaglutide 2.4 mg vs. Placebo | 68 wk | %change BW baseline to 68 wk & loss of at least 5% BW at 68 wk | Y | Y | Unknown | Monthly | Y | Food logs/diaries | Monthly* |
| Iijima et al, 2023 (150) | Semaglutide vs. Liraglutide | n=32, age > 20, T2DM on liraglutide 0.6 mg or 0.9 mg | 1:1 semaglutide vs. dulaglutide | 26 wk | %change HbA1c baseline to 26 wk | Y | Y | Unknown | Unknown | N | n/a | n/a |
| O'neil et al, 2018 (152) | Semaglutide vs. Liraglutide | n=957, BMI >/=30, no T2DM | 6:1, stratified by sex, semaglutide (0.5 mg, 0.1 mg, 0.2 mg, 0.3 mg, 0.4 mg) or liraglutide (3.0mg) vs. Placebo | 59 wk | %change BW baseline to 52 wk | Y | Y | Experienced practitioner | Every 4 wks | N | n/a | n/a |
| Rubino et al, 2022 (153) | Semaglutide vs. Liraglutide | n=338, BMI >/= 30 or 27 with 1 obesity-related comorbidity | 3:1:3:1 semaglutide 2.4 mg vs. Placebo, liraglutide 3.0 vs. Placebo | 68 wk | %change BW baseline to 68 wk | Y | Y | Experienced practitioner | Every 4-6 wks | N | n/a | n/a |
| Aronne et al, 2024 (26) | Tirzepatide | n=783, BMI >/= 30 or 27 with 1 obesity-related comorbidity | 1:1 tirzepatide 10 mg or 15 mg vs. Placebo | 88 wk | %change BW baseline to wk 36, then wk 88 | Y | Y | Experienced practitioner | Every 4 wks | N | n/a | n/a |
| Garvey et al, 2023 (24) | Tirzepatide | n=1514, BMI >/= 27, HbA1c 7-10% | 1:1 tirzepatide 10 mg or 15 mg vs. Placebo | 72 wk | %change BW baseline to 72 wk & loss of at least 5% BW at 72 wk | Y | Y | RDN or similarly qualified health care professional | Weekly | Y | Food logs/diaries | * |
| Heise et al, 2023 (41) | Tirzepatide | n=117, T2DM >/= 6 months, treated with lifestyle advice or stable metformin | 3:3:2 tirzepatide 15mg, semaglutide 1 mg or placebo | 28 wk | Measurements of body composition, appetite, and energy intake were performed as secondary assessments | N | N | n/a | n/a | Y | Ad libitum food intake during a 45-minute buffet-style lunch | Baseline & end of study |
| Jastreboff et al, 2022 (23) | Tirzepatide | n=2539, BMI >/= 30 or 27 with 1 obesity-related comorbidity | 1:1:1:1 tirzepatide 5 mg, 10 mg, 15 mg, vs. Placebo | 72 wk | %change BW baseline to 72 wk & loss of at least 5% BW at 72 wk | Y | Y | RDN or similarly qualified health care professional | Every 4 wks during dose escalation and then at every 12wks through 72 wks | N | n/a | n/a |
| Malhotra et al, 2024 (28) | Tirzepatide | n= 234 (trial 1), n= 235 (trial 2), BMI >/= 30, or >/= 27 in Japan; moderate-to-severe obstructive sleep apnea (AHI >/= 15 events per hour) | 1:1 tirzepatide vs. Placebo | 52 wk | Change in AHI from baseline to 52 wk | Y | Y | Unknown | Every 4 wks | N | n/a | n/a |
| Wadden et al, 2023 (25) | Tirzepatide | n=806, BMI >/= 27 with 1 obesity-related comorbidity or BMI >/=30 | 12 wk intensive lifestyle intervention lead-in period, if >/= 5% BW reduction, 1:1 to tirzepatide 10 mg or 15 mg vs. Placebo | 84 wk | %change BW baseline to 72 wk & loss of at least 5% BW at 72 wk | Y | Y | RDN or similarly qualified health care professional | Every 12 wks | Y | Food logs/diaries | Monthly |
| Zhao et al, 2024 (27) | Tirzepatide | n=210, BMI >/= 28 or >/= 24 with at least 1 obesity-related comorbidity with at least 1 reported unsuccessful dietary effort to lose wt | 1:1:1 tirzepatide 10 mg, 15 mg, vs. Placebo | 52 wk | %change BW baseline to 52 wk and proportion individuals losing >/= 5% baseline BW to 52 wk | Y | Y | RDN or similarly qualified health care professional | Weekly | N | n/a | n/a |
| AHI, apnea-hypopnea index; AMA, American Medical Association; BES, Binge Eating Scale; BOLD, blood oxygen level–dependent; BW, body weight; COEQ, Control of Eating Questionnaire; HbA1c, glycated hemoglobin; IBT, intensive behavioral therapy; NAFLD, nonalcoholic fatty liver disease; NASH, nonalcoholic steatohepatitis; OA, osteoarthiritis; OGTT, oral glucose tolerance test; RDN, Registered Dietitian Nutritionist; SF-36v2, Short Form-36 version 2; T2DM, type 2 diabetes mellitus; VAT, visceral adipose tissue; Wk, week; Wt, weight; Yr, year.  * The manuscript did not state explicit frequency of intake recording; thus an assumption was made that the intake method was collected at each study visit. | | | | | | | | | | | | |

Supplemental Table 2 Overview of SUSTAIN Clinical Trials Involving Semaglutide

|  | **Comparator Group** | **Study Duration** | **Primary outcome measured** | **Outcomes** | **Nutritional Counseling reported (Y/N)** | **Physical Activity Counseling reported (Y/N)** | **Dietary Intake Recorded (Y/N)** | **Diet Quality or Food Pattern Outcomes (Y/N)** | **Food Craving/Appetite Behavior Assessed (Y/N)** |
| --- | --- | --- | --- | --- | --- | --- | --- | --- | --- |
| SUSTAIN 1 [109] | placebo | 30 wk | %change HbA1c | -1.45% (0.5mg), -1.55% (1.0mg) vs. -0.02% placebo | N | N | N | N | N |
| SUSTAIN 2 [111] | oral sitagliptin and placebo | 56 wk | %change HbA1c | -1.3% (0.5mg), -1.6% (1.0mg) vs. -0.5% sitagliptin | N | N | N | N | N |
| SUSTAIN 3 [154] | exenatide ER 2.0mg | 56 wk | %change HbA1c | mean decrease -1.5% in semaglutide vs. 0.9% exenatide ER | N | N | N | N | N |
| SUSTAIN 4 [112] | insulin glargine | 30 wk | %change HbA1c | -1.21% (0.5mg), -1.64% (1.0mg) vs. -0.83 insulin glargine | N | N | N | N | N |
| SUSTAIN 5 [113] | placebo | 30 wk | %change HbA1c | -1.4% (0.5mg), -1.8% (1.0mg) vs. -0.1% placebo | N | N | N | N | N |
| SUSTAIN 6 [104] | placebo | 104 wk | primary composite outcome was the first occurrence of death from cardiovascular causes, nonfatal myocardial infarction (including silent), or nonfatal stroke | 6.6% in semaglutide group vs. 8.9% in placebo group | N | N | N | N | N |
| SUSTAIN 7 [128] | dulaglutide 0.76mg, 1.5 mg | 40 wk | %change HbA1c | -1.5% (0.5mg) vs. -1.1% dulaglutide (0.75mg), -1.8% (1.0mg) vs. -0.41% (dulaglutide 1.5mg) | N | N | N | N | N |
| SUSTAIN 8 [129] | canagliflozin 300 mg (daily) and placebo | 52 wk | %change HbA1c | -1.5% semaglutide vs. -1.0% canagliflozin | N | N | N | N | N |
| SUSTAIN 8 DXA [126] | canagliflozin 300 mg (daily) and placebo | 52 wk | Change from baseline to 52 wk in total fat mass (kg) | -3.4 kg semaglutide vs. -2.6 kg canagliflozin | N | N | N | N | N |
| SUSTAIN 9 [115] | placebo | 30 wk | %change HbA1c | -1.5% semaglutide vs. -0.1% placebo | N | N | N | N | N |
| SUSTAIN 10 [151] | liraglutide | 30 wk | %change HbA1c | -1.7% semaglutide vs. -1.0% liraglutide | N | N | N | N | N |
| SUSTAIN 11 [121] | Insulin Aspart 100 U/mL | 52 wk | %change HbA1c | -1.5% semaglutide vs. -1.2% insulin aspart | N | N | N | N | N |
| SUSTAIN CHINA [122] | oral sitagliptin and placebo | 30 wk | %change HbA1c | -1.4% (0.5mg), -1.7% (1.0mg), vs. -0.9% sitagliptin | N | N | N | N | N |
| SUSTAIN FORTE [123] | semaglutide 2.0mg vs. 1.0mg | 40 wk | %change HbA1c | -2.2% (2.0mg) vs. -1.9% (1.0mg) | N | N | N | N | N |
| HbA1c, glycated hemoglobin; Wk, week. | | | | | | | | | |

Supplemental Table 3 Summary of SURPASS Clinical Trials involving Tirzepatide

|  | **Sample Size & Population Characteristics** | **Comparator Group** | **Study Duration** | **Primary outcome measured** | **Outcomes** | **Nutritional Counseling reported (Y/N)** | **Physical Activity Counseling reported (Y/N)** | **Dietary Intake Recorded (Y/N)** | **Diet Quality Outcomes (Y/N)** | **Food Craving/Appetite Behavior Assessed (Y/N)** |
| --- | --- | --- | --- | --- | --- | --- | --- | --- | --- | --- |
| SURPASS 1 [138] | n=705, HbA1c 7.0-9.5%, BMI >/= 23, T2DM inadequately controlled with diet and exercise alone | placebo | 40 wk | %change in HbA1c | -1.87% (5mg), -1.89% (10mg), -2.07% (15 mg) vs., +0.04% placebo | N | N | N | N | N |
| SURPASS 2 [155] | n=1879, BMI >/= 25, HbA1c 7.0-10.5%, T2DM inadequately controlled with metformin of >/= 1500 mg/day | semaglutide 1 mg | 40 wk | %change in HbA1c | -2.01% (5mg), -2.24% (10mg), -2.30% (15mg) vs. -0.15% semaglutide | N | N | N | N | N |
| SURPASS 3 [143] | n=1444, BMI >/= 25, HbA1c 7.0-10.5%, T2DM on stable treatment metformin, with or without sodium-glucose co-transporter-2 medications | insulin degludec | 52 wk | %change in HbA1c | -1.93% (5mg), -2.20% (10mg), -2.37% (15mg) vs. -1.34% insulin degludec | N | N | N | N | N |
| SURPASS 4 [141] | n=2002, BMI >/=25, HbA1c 7.5-10.5%, T2DM, established cardiovascular disease or a high risk of cardiovascular event | glargine (100 U/mL) | 52 wk | %change in HbA1c | -2.44% (5mg), -2.43% (10mg), -2.58% (15mg) vs. -1.44% insulin glargine | N | N | N | N | N |
| SURPASS 5 [139] | n=475, BMI >/= 23, HbA1c 7.0-10.5%, T2DM, stable doses once daily insulin glargine with or without metformin >/= 1500 mg/d | placebo | 40 wk | %change in HbA1c | -2.40% (10mg), -2.34% (15mg) vs. -0.86% placebo | N | N | N | N | N |
| SURPASS 6 [140] | n=1428, BMI 23-45, HbA1c 7.5-11%, T2DM inadequately controlled with basal insulin, with or without oral anti-diabetic agents | insulin lispro | 52 wk | %change in HbA1c | mean change pooled tirzepatide doses -2.1% vs. -1.1% insulin lispro | N | N | N | N | N |
| SURPASS J-COMBO [133] | n=443, age > 20, BMI>/=23, HbA1c 7.0-11% inadequately controlled T2DM on oral anti diabetic agents’ monotherapy for at least 3 months | baseline oral anti-diabetic medications without tirzepatide | 52 wk | safety and tolerability during 52 wk treatment | Treatment-emergent adverse events 125 [84%] of 148 in 15 mg group vs. 109 [74%] of 148 and 109 [74%] of 147 in 5 mg and 10 mg group, respectively | N | N | N | N | N |
| SURPASS J-mono [144] | n=636, age > 20, BMI >/= 23, HbA1c 7.0-10.0%, T2DM, diet and exercise control or monotherapy anti-diabetic agent | dulaglutide 0.75 mg | 52 wk | %change in HbA1c | -2.4% (5mg), -2.6% (10mg), -2.8% (15mg) vs. -1.3% dulaglutide | N | N | N | N | N |
| SURPASS J-mono sub study [182] | n=48, age > 20, BMI >/= 23, HbA1c 7.0-10.0%, T2DM, diet and exercise control or monotherapy anti-diabetic agent | dulaglutide 0.75 mg | 52 wk | postprandial metabolic characteristics of appetite & AUC 0-6h after dose for plasma glucagon, serum glucose, insulin, C-peptide, and triglycerides. | mean reduction in glucose AUC of −7.5 mmol·h/L (5mg), -11.2 mmol·h/L (10mg), -14.2 mmol·h/L (15mg) vs. dulaglutide | N | N | N | N | Y |
| SURPASS-AP-COMBO [146] | n=917, BMI >/= 23, HbA1c 7.5-11.0%, T2DM inadequately controlled insulin native, on metformin with or without sulphonylurea. | insulin glargine | 40 wk | %change in HbA1c | -2.24% (5mg), -2.44% (10mg), -2.49% (15mg) vs. -0.95% insulin glargine | N | N | N | N | N |
| HbA1c, glycated hemoglobin; T2DM, type 2 diabetes mellitus; wk, week. | | | | | | | | | | |

Supplemental Table 4 Summary of Semaglutide Treatment Effect in People with Obesity (STEP) Trials

|  | **Sample Size & Population Characteristics** | **Comparator Group** | **Study Duration** | **Primary outcome measured** | **Outcomes** | **Nutritional counseling reported (Y/N)** | **Physical activity counseling reported (Y/N)** | **Dietary Intake Recorded (Y/N)** | **Diet Quality Outcomes (Y/N)** | **Food Craving/Appetite Behavior Assessed (Y/N)** |
| --- | --- | --- | --- | --- | --- | --- | --- | --- | --- | --- |
| STEP 1 [6] | n=1961, BMI >/= 30 or 27 with 1 obesity-related comorbidity | 2:1 semaglutide 2.4 mg vs. placebo | 68 wk | %change BW baseline to 68 wk & loss of at least 5% BW at 68 wk | -Mean weight loss: **14.9%** with semaglutide 2.4 mg vs. 2.4% with placebo at 68 weeks.  - ≥5% weight loss: 86.4% (semaglutide) vs. 31.5% (placebo). | Y | Y | Y | N | N |
| STEP 2 [7] | n=1210 ,BMI >/= 27, T2DM | 1:1:1, 2.4 mg, 1.0 mg., vs. placebo | 68 wk | %change BW baseline to 68 wk & loss of at least 5% BW at 68 wk | -Mean weight loss: **9.6%** with semaglutide 2.4 mg vs. 3.4% with placebo at 68 weeks.  - ≥5% weight loss: 68.8% (semaglutide) vs. 28.5% (placebo).  - HbA1c reduction: 1.6% (semaglutide) vs. 0.4% (placebo). | Y | Y | Y | N | N |
| STEP 3 [8] | n=611, BMI >/= 30 or 27 with 1 obesity-related comorbidity | 2:1 semaglutide 2.4 mg vs. placebo | 68 wk | %change BW baseline to 68 wk & loss of at least 5% BW at 68 wk | -Mean weight loss: **16.0%** with semaglutide 2.4 mg vs. 5.7% with placebo at 68 weeks.  -≥5% weight loss: 86.6% (semaglutide) vs. 47.6% (placebo). | Y | Y | Y | N | N |
| STEP 4 [124] | n=902, BMI >/= 30 or 27 with 1 obesity-related comorbidity | all open label semaglutide until wk 20; 2:1 semaglutide 2.4 mg vs. placebo after wk 20 | 68 wk | %change BW wk 20 to wk 68 | -Continued semaglutide: additional 7.9% weight loss from week 20 to 68.  - Switched to placebo: **6.9% weight regain**. Demonstrated importance of ongoing treatment for weight maintenance. | Y | Y | Y | N | N |
| STEP 5 [125] | n=304, BMI >/= 30 or 27 with 1 obesity-related comorbidity | 1:1 semaglutide 2.4 mg vs. placebo | 104 wk | %change BW baseline to 104 wk & loss of at least 5% BW at 104 wk | - Mean weight loss: **15.2%** with semaglutide 2.4 mg vs. 2.6% with placebo at 104 weeks.  - ≥5% weight loss: 77.1% (semaglutide) vs. 34.4% (placebo). | Y | Y | Y | N | Y |
| STEP 5 subgroup [9] | n=174, BMI >/= 30 or 27 with 1 obesity-related comorbidity | 1:1 semaglutide 2.4 mg vs. placebo | 104 wk | association between changes in COEQ domain scores and BW | - Mean body weight changes were **-14.8%** (semaglutide) and -2.4% (placebo). Scores significantly improved with semaglutide vs. placebo for Craving Control and Craving for Savory domains at weeks 20, 52, and 104 (p < 0.01); for Positive Mood and Craving for Sweet domains at weeks 20 and 52 (p < 0.05); and for hunger and fullness at week 20 (p < 0.001). | Y | Y | Y | N | Y |
| STEP 6 [10] | n=401, BMI >/= 35 or 27 with >/= 2 obesity-related comorbidity | 4:1:2:1 semaglutide 2.4 mg vs placebo or semaglutide 1.7 mg vs placebo | 68 wk | %change BW baseline to 68 wk & loss of at least 5% BW at 68 wk | - Mean weight loss: **13.2%** with semaglutide 2.4 mg vs. 2.1% with placebo at 68 weeks.  - ≥5% weight loss: 83% (semaglutide) vs. 21% (placebo). | Y | Y | Y | N | N |
| STEP 6 subgroup [11] | n=401, BMI >/= 35 or 27 with >/= 2 obesity-related comorbidity | 4:1:2:1 semaglutide 2.4 mg vs placebo or semaglutide 1.7 mg vs placebo | 68 wk | baseline to wk 68 in the Impact of Weight on Quality of Life–Lite Clinical Trials Version Physical Function score and the SF-36v2 Physical Functioning score | - Impact of Weight on Quality of Life–Lite Clinical Trials Version physical score function score improved 3.4 ETD [95% CI (-0.1, 6.9) with 2.4mg vs. placebo, and 2.0 ETD [95% CI (-2.0, 6.0) in 1.7 mg vs. placebo.  - SF‑36v2 Physical Functioning improved 1.2 ETD [95% CI (0.1, 2.2) in 2.4 mg vs. placebo, and 0.3 ETD [95% CI -1.0, 1.5] in 1.7 mg vs. placebo | Y | Y | Y | N | N |
| STEP 7 [127] | n=375, BMI >/= 30 or 27 with 1 obesity-related comorbidity, T2DM if diagnosed >/= 180d prior and on stable treatment | 2:1 semaglutide 2.4 mg vs. placebo | 44 wk | %change BW baseline to 44 wk & loss of at least 5% BW at 44 wk | - Mean weight loss: **12.1%** with semaglutide 2.4 mg vs. 3.6% with placebo at 44 weeks.  - ≥5% weight loss: 85.4% (semaglutide) vs. 26.8% (placebo). | Y | Y | Y | N | N |
| STEP 8 [153] | n=338, BMI >/= 30 or 27 with 1 obesity-related comorbidity | 3:1:3:1 semaglutide 2.4 mg vs. placebo, liraglutide 3.0 vs. placebo | 68 wk | %change BW baseline to 68 wk | - Mean weight loss: **15.8%** with semaglutide vs. 6.4% with liraglutide at 68 weeks.  - ≥5% weight loss: 86.6% (semaglutide) vs. 47.6% (liraglutide).  - Demonstrated superior efficacy of semaglutide over liraglutide. | Y | Y | N | N | N |
| STEP 9 [12] | n=407, BMI >/= 30, clinical diagnosis of knee osteoarthritis according to the American College of Rheumatology criteria with pain related to knee osteoarthritis on Western Ontario and McMaster Universities OA Index pain score >/= 40 | 2:1 semaglutide 2.4 mg vs. placebo | 68 wk | %change in BW and the change in Western Ontario and McMaster Universities OA Index pain score from baseline to 68 wk | -Mean weight loss: **13.7%** with semaglutide vs. 3.2% with placebo at 68 wks.  -Improvement of -41.7 points vs. -27.5 with placebo Western Ontario and McMaster Universities OA Index score. | Y | Y | N | N | N |
| STEP 10 [13] | n=138, BMI >/= 30, and prediabetes having at least 1: HbA1c 6.0-6.4% or fasting plasma glucose 5.5-6.9 mmol/L | 2:1 semaglutide 2.4 mg vs. placebo | 52 wk | %change BW baseline to 52 wk and proportion of participants who reverted to normoglycemia | -Mean weight loss: **13.9%** in semaglutide vs. 2.7% in placebo.  -81% of individuals in semaglutide group returned to normoglycemia at wk 52 than 14% in placebo, OR= 19.8 [95% CI 8.7-45.2, p<0.0001) | Y | Y | N | N | N |
| STEP-HFpEF [14] | n=529, BMI >/=30, left ventricular ejection fraction >/= 45%, NY Heart association functional class II, III, or IV symptoms, KCCQ-CSS </=90 | 1:1 semaglutide 2.4 mg vs. placebo | 52 wk | change in the KCCQ-CSS and the % change in BW from baseline to 52 wk | -The mean change in the KCCQ-CSS was 16.6 points with semaglutide vs. 8.7 points with placebo.  - The mean percentage change in body weight was **−13.3%** with semaglutide and −2.6% with placebo | N | N | N | N | N |
| STEP-HFpEF DM [131] | n=616, BMI >/=30, left ventricular ejection fraction >/= 45%, HbA1c </=10%, T2DM | 1:1 semaglutide 2.4 mg vs. placebo | 52 wk | change in the KCCQ-CSS and the % change in BW from baseline to 52 wk | - The mean change in the KCCQ-CSS was 13.7 points with semaglutide vs. 6.4 points with placebo - The mean percentage change in body weight was −9.8% with semaglutide and −3.4% with placebo | N | N | N | N | N |
| BW, body weight; COEQ, Control of Eating Questionnaire; DM, diabetes mellitus; ETD, estimated treatment difference; HbA1c, glycated hemoglobin; KCCQ-CSS, Kansas City Cardiomyopathy Questionnaire–Clinical Summary Score; OA: osteoarthritis; SF-36v2, Short Form-36 version 2; STEP, Semaglutide Treatment Effect in People with Obesity; T2DM, type 2 diabetes mellitus; wk, week. | | | | | | | | | | |

Supplemental Table 5 Summary of SURMOUNT (Tirzepatide) Trials

|  | **Sample Size & Population Characteristics** | **Comparator Group** | **Study Duration** | **Primary outcome measured** | **Outcomes** | **Nutritional counseling reported (Y/N)** | **Physical activity counseling reported (Y/N)** | **Dietary Intake Recorded (Y/N)** | **Diet Quality Outcomes (Y/N)** | **Food Craving/ Appetite Behavior Assessed (Y/N)** |
| --- | --- | --- | --- | --- | --- | --- | --- | --- | --- | --- |
| SURMOUNT 1 [19] | n=2539, BMI >/= 30 or 27 with 1 obesity-related comorbidity | 1:1:1:1 tirzepatide 5 mg, 10 mg, 15 mg, vs placebo | 72 wk | %change BW baseline to 72 wk & loss of at least 5% BW at 72 wk | Mean wt loss at 72 wks: **15.0%** (5 mg), **19.5%** (10 mg), and **20.9%** (15 mg) vs. 3.1% with placebo.  - ≥5% wt loss achieved by 85–91% with tirzepatide vs. 35% with placebo | Y | Y | N | N | N |
| SURMOUNT 2 [20] | n=1514, BMI >/= 27, HbA1c 7-10% | 1:1 tirzepatide 10 mg or 15 mg vs placebo | 72 wk | %change BW baseline to 72 wk & loss of at least 5% BW at 72 wk | Mean wt loss at 72 wks: **12.8%** (10 mg) and **14.7%** (15 mg) vs. 3.2% with placebo.  - ≥5% wt loss achieved by 79–83% with tirzepatide vs. 32% with placebo.  - Significant improvements in HbA1c and other cardiometabolic parameters. | Y | Y | Y | N | N |
| SURMOUNT 3 [21] | n=806, BMI >/= 27 with 1 obesity-related comorbidity or BMI >/=30 | 12 wk intensive lifestyle intervention lead-in period, if >/= 5% BW reduction, 1:1 to tirzepatide 10 mg or 15 mg vs. placebo | 84 wk | %change BW baseline to 72 wk & loss of at least 5% BW at 72 wk | Additional mean wt loss at 72 wks: **18.4%** with tirzepatide vs. 2.5% with placebo.  - ≥5% additional wt loss achieved by 87.5% with tirzepatide vs. 16.5% with placebo.  - Total mean wt loss from baseline: approximately 24.3% with tirzepatide. | Y | Y | Y | N | N |
| SURMOUNT 4 [22] | n=783, BMI >/= 30 or 27 with 1 obesity-related comorbidity | 1:1 tirzepatide 10 mg or 15 mg vs placebo | 88 wk | %change BW baseline to wk 36, then wk 88 | Continued tirzepatide for 52 wks: additional 5.5% wt loss; total mean wt loss from baseline: 25.3%.  - Switched to placebo: 14.0% wt regain.  - 89.5% of those continuing tirzepatide maintained ≥80% of prior wt loss vs. 16.6% with placebo. | Y | Y | N | N | N |
| SURMOUNT-CN [23] | n=210, BMI >/= 28 or >/= 24 with at least 1 obesity-related comorbidity with at least 1 reported unsuccessful dietary effort to lose wt. | 1:1:1 tirzepatide 10 mg, 15 mg, placebo | 52 wk | %change BW baseline to 52 wk and proportion individuals losing >/= 5% baseline BW to 52 wk | The mean change in BW at wk 52 was **-13.6%** (95% CI, -15.8% to -11.4%) with tirzepatide 10 mg, **-17.5%** (95% CI, -19.7% to -15.3%) with tirzepatide 15 mg, and -2.3% with placebo.  -The percentage of participants achieving body wt reductions of 5% or greater was 87.7% with tirzepatide 10 mg, 85.8% with tirzepatide 15 mg, and 29.3% with placebo (p < .001 for comparisons with placebo) | Y | Y | N | N | N |
| SURMOUNT-OSA [24] | n= 234 (trial 1), n= 235 (trial 2), BMI >/= 30, or >/= 27 in Japan; moderate-to-severe obstructive sleep apnea (AHI >/= 15 events per hour) | 1:1 tirzepatide vs placebo | 52 wk | change in AHI from baseline to 52 wk | Mean change in AHI at wk 52 was −25.3 events per hour (95% confidence interval [CI], −29.3 to −21.2) with tirzepatide and −5.3 events per hour (95% CI, −9.4 to −1.1) with placebo | Y | Y | N | N | N |
| **AHI**, apnea–hypopnea index; **BW**, body weight; **HbA1c**, glycated hemoglobin; **Wk**, week; Wt, weight. | | | | | | | | | | |
